# CD8^+^ T–NK cell crosstalk establishes preemptive immunosurveillance to eliminate antigen–escape tumors

**DOI:** 10.3389/fimmu.2025.1593913

**Published:** 2025-09-22

**Authors:** Roman V. Uzhachenko, Salvador González Ochoa, Thanigaivelan Kanagasabai, Harshana Rajakaruna, Menaka C. Thounaojam, Maria Teresa P. de Aquino, Tanu Rana, Lino Costa, Alexander Terekhov, William H. Hofmeister, Thomas J. Sayers, Claude Boyer, Alla V. Ivanova, J. Shawn Goodwin, Anne-Marie Schmitt-Verhulst, Anil Shanker

**Affiliations:** ^1^ Department of Biochemistry, Cancer Biology, Neuroscience and Pharmacology, Meharry Medical College School of Medicine, Nashville, TN, United States; ^2^ The Office for Research and Innovation, Meharry Medical College, Nashville, TN, United States; ^3^ Department of Biomedical Sciences, Meharry Medical College School of Graduate Studies, Nashville, TN, United States; ^4^ Center for Laser Applications, University of Tennessee Space Institute, Tullahoma, TN, United States; ^5^ Basic Research Program, Leidos Biomedical Research, Inc., Frederick National Laboratory for Cancer Research, Frederick, MD, United States; ^6^ Cancer and Inflammation Program, National Cancer Institute-Frederick, Frederick, MD, United States; ^7^ Centre d’Immunologie de Marseille-Luminy, Aix Marseille Université UM2, Institut National de la Santé et de la Recherche Médicale U1104, Centre National de la Recherche Scientifique UMR7280, Marseille, France

**Keywords:** CD8^+^ T lymphocytes, T cell–NK cell cooperativity, tumor immune escape, antigen-loss variants, adoptive cell transfer, cancer immunotherapy, membranous tunneling nanotubes, preemptive immunosurveillance

## Abstract

**Background and objective:**

Tumor antigen–escape variants undermine immunotherapy by subverting lymphocyte effector functions and reshaping tumor–immune dynamics. It is essential to delineate functional interplay within immune networks during tumor progression. We investigated whether homeostatic crosstalk between CD8^+^T cells and natural killer (NK) cells preempts tumor antigen–escape.

**Methods:**

Adoptive CD8^+^T cell transfers were administered before (D_–7_, homeostatic pre-priming) or after (D_+1_) tumor establishment in *Rag1^−/−^* and *Rag1^−/−^γc^−/−^* mice. Antigen presentation, immune activation, proliferation, cytotoxicity, and memory were quantified by flow cytometry, live bioluminescence and confocal imaging. Monoculture, co-culture, and a 3D silica nanofiber carpet mimicking basement-membrane-like topography modeled intercellular interactions. Signaling arrays and motion metrics (Speed-Distance Index, deceleration) were conducted. Human ligand–receptor pairs engaged in CD8^+^T–NK crosstalk were probed *in silico*.

**Results and discussion:**

Pre-tumor D_–7_ CD8^+^T cell transfer completely suppressed antigen–escape tumors with NK cells as major effectors showing elevated CD25, CD69, CD107a, and GzmB, marking activated and effector phenotype, and promoting central-memory CD62L⁺CD44⁺CD8⁺T_CM_ precursors. By contrast, post-tumor D_+1_–transferred CD8^+^T cells allowed emergence of tumor variants resistant to antigen-specific cytolysis as assessed on day 25, despite those T cells retaining higher intrinsic cytotoxic capacity than the D_–7_ T cell cohort. Mechanistically, CD8^+^T and NK cells formed stable contacts through pseudopodial intercellular nanotubes enabling bidirectional membrane exchange and signaling *via* STAT, Akt, and mTOR pathways, augmenting NK effector function and promoting CD8^+^T_CM_ differentiation. *In silico* analysis identified human ligand–receptor pairs engaged in CD8^+^T–NK adhesion, stimulatory and regulatory axes, including CD200–CD200R, PD-L1–PD-1, and CD18/CD11a–DNAM-1 (CD226). Together, data support a three-phase model of preemptive immunosurveillance initiated by early CD8⁺T–NK crosstalk.

**Conclusion:**

Homeostatic conditioning and effector cooperativity between CD8^+^T and NK cells protect against tumor immune escape. The findings uncover a mechanistic axis of preemptive immunosurveillance that lays the foundation for next-generation preventive immunotherapies to control antigen–escape tumors.

## 1 Introduction

The immune system is a highly dynamic network of specialized cells whose functions are governed by intricate regulatory feedback loops. A key challenge in immunology is understanding how lymphocyte interactions adapt in tumor-afflicted tissues, where the immunosuppressive inflammatory environment often undermines antitumor lymphocytes and the crosstalk between intratumoral immune cells (1, 2). Even genetically engineered antitumor T cells from a cancer patient, bolstered by immune checkpoint blockade antibodies, have failed to increase the post-metastasis 5-year relapse-free survival rate in most malignancies (< 25%) other than leukemia and desmoplastic melanoma (3). Further limiting the effectiveness of engineered TCR T cell therapies are issues such as TCR chain mispairing and the generation of self-reactive TCR heterodimers, which can lead to lethal off-target responses (4, 5). In addition, the extreme sensitivity and specificity with which the TCR-transduced T cells recognize tumor cells could enhance the antigenic drift in the “immunoedited” tumor variants, involving distinct cell germline genes (6). These complexities with intrinsic safety risks curtail the efficacy of current T cell therapies and may jeopardize the outcomes of the personalized mutanome vaccines aiming to elicit robust antitumor T cell responses (7).

Furthermore, tumor endogenous strategies can shape disease progression and promote the emergence of antigen–escape variants, impacting the T cell response due to their lack of immunogenicity, which is considered an effective mechanism used by "cold" tumor cells to evade the immune recognition (8, 9). During the effector phase, cells such as CD4^+^T lymphocytes, dendritic cells, and even stromal cells play crucial roles in sustaining CD8^+^T cell responses (10, 11). However, it is poorly acknowledged how CD8^+^T cells encounter challenges in mounting effective responses against tumor antigens during the initial encounter and beyond the effector phase through the generation of memory.

Therefore, it is imperative to delve into the complex immunological mechanisms and cellular interactions underlying the development of a response to tumor antigen–escape variants. This knowledge is fundamental in devising targeted therapies that can support the immune system in managing and controlling the evolving tumor variants. Using a tumor model expressing the cancer–germline self-antigen P1A encoded by the X-linked gene *Trap1a*, a model akin to human cancer–germline counterparts such as *MAGE* (12, 13), we discovered a unique tumor-localized phenomenon, where CD8^+^T cells play an essential role in activating NK cells (14, 15). This study investigates the biological significance of homeostatic immune priming and mechanisms underlying interactions between CD8^+^T lymphocytes and NK cells to provide effective immunosurveillance against tumor antigen–escape variants. The results demonstrate that preemptive adoptive T cell transfer not only prevents tumor development but also drives complete rejection of emerging antigen–escape variants by eliciting a cooperative effector program involving CD8^+^T and NK cells.

## 2 Materials and methods

### 2.1 Mice

BALB/c, *Rag1* ^−/−^B6, or *Rag1* ^−/−^B10.D2-*Hc^1^ H2^d^ H2-T18^c^*/nSnJ mice (6–8 weeks) were purchased from Harlan Laboratories or The Jackson Laboratory. Mice heterozygous for H-2L*^d^*/P1A_35-43_-specific TCR transgene were maintained on the *Rag1* ^−/−^B10.D2 background (TCRP1A-B10.D2 *Rag1* ^−/−^) and genotyped as described previously (16). Mice deficient for the common cytokine receptor γ-chain (γc^−/−^) (courtesy J. P. Di Santo, Institut Pasteur, Paris, France) were crossed with *Rag1* ^−/−^B10.D2 mice to establish a *Rag1* ^−/−^γ*c* ^−/−^B10.D2 line. Mice *Gzmb-Tom-KI/KI* was produced by homologous recombination to express GZMB-Tomato registered as EM:05732 at the European Mouse Mutant Archive (EMMA), crossed with *Rag1* ^−/−^B10.D2 mice and genotyped as described previously (17). Mice were bred and housed in a pathogen-free environment in the animal facility of Centre d’Immunologie de Marseille-Luminy (Marseille, France), National Cancer Institute (NCI)-Frederick, or Meharry Medical College. The studies complied with the protocols approved by the Institutional Animal Care and Use Committee regulations and were in accordance with the procedures outlined in the National Institutes of Health Guide for the Care and Use of Laboratory Animals and French and European directives.

### 2.2 Cell lines

Mastocytoma P815 (H-2*^d^*) sublines, namely, P511 expressing P1A and P1.204 deficient in P1A but similar in co-stimulatory or MHC expression characteristics (18) (courtesy B. Van den Eynde, Ludwig Institute for Cancer Research, Brussels, Belgium). Tumor cells were maintained in 10% FCS-supplemented standard RPMI-1640 medium and authenticated regularly with reference stocks to ensure fidelity; sterility and mycoplasma testing were also performed routinely. Low-passage (**<** 5) tumor cell cultures were used for the experiments.

### 2.3 Tumor establishment

Mastocytoma sublines (P511 and P1.204) were transplanted individually (1 x 10^6^) or mixed (1 x 10^6^ each) s.c. between brachial and inguinal LN. Luciferase-expressing tumor cell clones, P511-Luc or P1.204-Luc, were obtained after transfection of P511 or P1.204 cells with vector pEGFPLUC (BD Biosciences/BD Clontech) by Lipofectamine 2000 (Invitrogen Life Technologies), followed by G418 selection. Tumors showed a well-localized and regularly shaped form. In some experiments, tumor transplantation was performed with cells isolated from the solid tumor mass excised after *in-vivo* growth of at least 14 days in *Rag1* ^−/−^ or 25 days in ^TCRP1A^CD8^+^T cell-transferred *Rag1* ^−/−^ mice using the Tumor Dissociation Kit, mouse (Cat# 130-096-730, Miltenyi Biotec). The tumor growth was monitored by measuring the two perpendicular diameters of the solid tumors every fourth day with Vernier calipers. Tumor size or survival was recorded for at least three months or until the tumor burden reached 400 mm^2^. Survival analysis was performed using GraphPad Prism v10.2.3 (GraphPad Software), and the statistical difference between the survival curves was analyzed using a log-rank test.

### 2.4 T cell adoptive transfers and animal imaging

Mice received an i.v. injection of naïve ^TCRP1A^CD8^+^T cells isolated from LN of ^TCRP1A^ *Rag1* ^−/−^B10.D2 mice in pre- and post-tumor establishment conditions. In other experiments, mice were shaved on the flanks and then injected s.c. with 1 x 10^6^ P511-Luc or P1.204-Luc individually or with a mixture of 1 x 10^6^ P511 plus 1x10^6^ P1.204-Luc on the same flank. Some mice received an i.v. injection of 2 x 10^6 TCRP1A^CD8^+^T cells 5 days earlier. Tumor growth was monitored by luciferase signal from luciferase-expressing cells, followed by bioluminescence imaging. After luciferin (3 mg/mouse, i.p. injection), the mice were anesthetized in a chamber flushed with a mixture of isofluorane (4% in air) and placed in the NightOwl LB981 (Berthold Technologies) under continuous anaesthetization (1.5-3% isofluorane in O_2_). First, a black-and-white photographic image was acquired using a 100-ms exposure. Next, 10 minutes after luciferin injection, the luminescent image was acquired using a 2-minute photon integration period with background subtraction (pixel binning 8x8). Quantification was performed using Berthold Technologies software. For visualizing endogenous Tomato fluorescence corresponding to the GzmB-tdTom (GzmB) protein, 1 x 10^6^ P1A^+^P511 cells were established s.c. in *Rag1^−/−^* B10.D2 or *Rag1^−/−^* GzmB-TomB10.D2 mice followed by an i.v. transfer of 3 x 10^6 TCRP1A^CD8^+^T cells on day 7. Tumor sections from mice euthanized after five days with or without T cell transfer were stained with NKp46 and CD8 antibodies and analyzed by confocal imaging.

### 2.5 Syngeneic dendritic cell differentiation and T cell activation

The generation of bone marrow-derived dendritic cells (DCs) was performed according to the published methodology in Machy P. et al. (19). Briefly, bone marrow cells were obtained from DBA/2 mice and cultured for three days in Dulbecco’s modified Eagle’s minimum essential medium (DMEM) supplemented with 10% fetal bovine serum (FBS), antibiotics, 2 mM glutamine, 2-mercaptoethanol 0.05 mM, and 30% conditioned medium from NIH3T3 cells containing granulocyte-macrophage colony-stimulating factor (GMCSF). The resulting cells were then diluted in 1:1 in the same medium (DMEM) and cultured for 10 days in non–tissue culture-treated 50 mm dishes. The non-adherent cells were washed and resuspended in supplemented RPMI-1640. After that, DCs were incubated overnight with 10^−7^ M P1A peptide (P1Ap) or media only as a control. Following the loading with P1A or media, the DCs were sub cultured in a 1:1 ratio with the CD8^+^T cells. Once T cells were fully activated (CD69^high^CD25^high^CD62L^low^) and purified using CD8^+^T (Cat# 130-104-075, Miltenyi Biotec), they were used for T cell transfers.

### 2.6 Cytotoxicity assay

The cytolytic assay was performed by incubating purified stimulated or unstimulated ^TCRP1A^CD8^+^T cells with P1A^+^P511 or P1A^−^P1.204 tumor target cells labeled with ^51^Cr (New England Nuclear, Boston, MA). Purified ^TCRP1A^CD8^+^T cells were stimulated *in vitro* for 3 days in 24-well plates with syngeneic wt T-depleted splenic APCs (10^6^) loaded with the relevant cognate P1A_35–43_ peptide (LPYLGWLVF) or an irrelevant lymphocytic choriomeningitis virus nucleoprotein NP_118–126_ peptide. Target cells (1 x 10^4^) were incubated with effector cells at 37°C, and the ^51^Cr release was assayed after 5.5 h.

### 2.7 T cell proliferation assay

The T cell divisions were quantified by flow cytometry using the intracytoplasmic stable 5-,6-carboxyfluorescein diacetate succinimidyl ester dye (CFSE) that exhibits sequential halving of the fluorescence intensity with each successive division. Purified ^TCRP1A^CD8^+^T cells were incubated for 10 min at 37 °C with 5 μM CFSE (Molecular Probes, Eugene, OR) and washed before *in-vitro* culture with indicated conditions or injection in mice.

### 2.8 CD8^+^T–NK cell co-culture

Cells were purified by negative selection from the LN or splenocyte fraction using CD8^+^T (Cat# 130-104-075, Miltenyi Biotec) or NK cell isolation kits (Cat# 130-115-818, Miltenyi Biotec) according to the protocol provided by the manufacturer. The purity was estimated at ~95% by FACS analysis with specific cell-typic Abs. Purified CD8^+^T cells were stimulated with PMA (50 ng/mL) and ionomycin (1 μg/mL) for 36 h in RPMI-1640 medium supplemented with 10% FBS and 1% antibiotic-antimycotic mixture (Gibco) at 37 °C and 5% CO_2_. Once ~80% of T cells were CD25^high^CD69^high^ by FACS, they were washed with PBS to remove residual PMA and ionomycin. Activated CD8^+^T cells were co-cultured with freshly isolated NK cells in a ratio 1:1 up to 36 h in a 3D silica nanofiber carpet plate or regular 24- or 96-well plates pre-coated with fibronectin (10 µg/mL) from bovine plasma (Sigma) for 1 h at 37 °C to increase intercellular contacts between CD8^+^T and NK cells as described previously (20). For controls, naïve or activated CD8^+^T and NK cells were cultured individually.

### 2.9 Live-cell 2D and 3D imaging of CD8^+^T–NK cell interactions and analysis of co-localization

Cells were imaged to obtain two-dimensional (2D) images in eight-well chambered cover glasses (Chambered Borosilicate Cover glass; Lab-Tek) coated with 10 µg/mL fibronectin (Sigma). For three-dimensional (3D) modeling, we prepared a scaffold by casting the polymer ϵ-polycaprolactone (PCL) (Sigma-Aldrich) into a femtosecond laser-machined glass template following the method of Rajput (21). The length, diameter, and density of nanofibers extracted from the base holes were controlled. Cast nanofibers were extracted from the template by thermally bonding a polycarbonate coverslip (Grace Bio-Labs HybriSlip, 22x22x0.25 mm) to the backside of the nanofibers pattern. The coverslip was then glued (Grace Bio-Labs SA-S-1L-SecureSeal 0.12 mm thick) to the bottom of a 35 mm culture dish (MaTek Corp., P35G-0-14C) in place of the glass cover slip. Several template patterns and nanofiber configurations were explored until a suitable nanofiber mat was achieved. Cells were imaged with a Nikon A1R laser scanning confocal microscope using excitation wavelengths of 488 and 561 with a 60x oil immersion objective (numerical aperture 1.4) and analyzed by Nikon Advanced Research Imaging Software. For a visualization of the intercellular contact formation, 5 x 10^5^ primary naïve or activated CD8^+^T cells were labeled with 2 μM DiD (3H-Indolium, 2-(5-(1,3-dihydro-3,3-dimethyl-1-octadecyl-2H-indol-2-ylidene)-1,3-pentenyl)-3,3-dimethyl-1-octadecyl-, perchlorate) according to the manufacturer’s instructions and co-incubated with 5 x 10^5^ naïve NK cells stained with 2 μM DiO (Benzoxazolium,3-octadecyl-2-[3-(3-octadecyl-2(3H)-benzoxazolylidene)-1-propenyl]-perchlorate) for 24 h in 8-well chambers or 1 x 10^6^ of each cell subset were added to the 3D nanofiber ϵ-polycaprolactone carpet surface. Time-lapse confocal microscopy was used for cell tracking and velocity analysis to monitor CD8^+^T cell interactions with NK cells in co-culture conditions. Individual CD8^+^T cells were manually tracked over time using ImageJ’s MTrackJ plugin. For each tracked cell, three behavioral phases were defined: Phase 1 (Ph1)—before contact with an NK cell, Phase 2 (Ph2)—during direct contact (as confirmed by membrane overlap or synapse-like contact), and Phase 3 (Ph3)—after contact ended. Velocities were calculated by dividing the total displacement during each phase by the corresponding time interval (μm/s). A deceleration ratio was computed as the average velocity during contact (Ph2) divided by the average velocity before contact (Ph1) for each cell. These ratios were used to assess changes in motility upon NK cell engagement. Cells were plotted in order of an increasing deceleration ratio, with the X-axis representing the cell index and the Y-axis showing the corresponding ratio. Finally, the analysis of co-localizing pairs was performed by Speed-Distance Index (SDI). The SDI interaction metric between NK and T cells at time t is defined as:


$$SDI_{\mathrm{NK}}{↔}_{T}(t)\frac{v_{\mathrm{rel}}(t)}{d_{\mathrm{NK,T}}(t)+\text{ϵ}}$$

Where:

-
$v_{rel}(t)=||{\vec{v}}_{NK}(t)-{\vec{v}}_{T}(t)||$ : Relative speed between the NK and T cells at time *t*
-
$d_{NK,T}(t)$ : Euclidean distance between the NK and T cells at time *t*
- *ϵ*: A small constant to avoid division by zero

### 2.10 Immunofluorescence staining

Cells were stained for immunofluorescence staining, following the FcγR-blocking, with various antibodies or control isotypes obtained as follows: fluorochrome-conjugated anti-mouse isotype or monoclonal antibodies against CD8, CD49b, NK1.1, NKp46, CD25, CD69, CD44, CD62L, PD-1, PD-L1, IL-15R, CD122, CD132, GzmB, FasL, H-60, MULT-1, pan-Rae-1, anti-human IgG Fc-APC, anti-mouse and anti-rabbit IgG-DyLight488 (Biolegend); Anti-NKG2D MI6 (eBioscience); primary antibodies to phosphorylated and total proteins of NFκB p65, mTOR, AKT, p42/p44, p38, SAPK/JNK, LCK, PI3K, JAK1-3, PTEN, TYK2, STAT1-6, and bulk NFAT1 (Cell Signaling); antibodies to phosphorylated NFAT1 (GeneTex); recombinant mouse IL-2 (eBioscience), recombinant mouse chimera IL-2/Fc protein was from Sigma. Intracellular cytokine staining (ICS) and PhosphoFlow assay were performed according to the manufacturer’s instructions (BD Biosciences) using mouse IFNγ-PE, IL-2-FITC, IL-4-PE antibodies (0.5 μL/100 μL permeabilization buffer) (Biolegend). To measure IL-2 capture from the culture medium, we exposed cells for 18–24 h to 100 p/mol recombinant mouse IL-2/Fc chimera protein and stained cells with anti-IgG Fc Abs according to the ICS protocol as described (22). Data were acquired on leukocyte gates as per forward and side scatters using a FACSCalibur, LSR-I flow cytometer (BD Biosciences), or Guava EasyCyte HT system (EMD Millipore) and analyzed using Cell-Quest (BD Biosciences) or FlowJo software v.10.9 (Treestar Inc).

### 2.11 Evaluation of proteins and phosphoproteins by flow cytometry

We implemented three distinct protocols of cell staining upon co-cultures. Each protocol served a specific purpose.

Protocol 1: *membrane (surface) immunostaining.* Cells were washed with D-PBS (1X) from Thermo Fisher, followed by incubation for 15 minutes with rat anti-mouse TruStain FcX™ (anti-mouse CD16/32) antibody (Biolegend) and Zombie Aqua™ Fixable Viability Kit (Biolegend) to discriminate between live and dead cells. After a single wash, staining was carried out for 30 minutes at room temperature, followed by a wash and fixation with fixation buffer (Biolegend) for 20 minutes at room temperature. Finally, cells were centrifuged at 300 G for 5 minutes and resuspended for flow cytometry using the Cytex Amnis CellStream benchtop flow cytometer (Cytek^®^ Biosciences).

Protocol 2: *membrane* & *intracellular immunostaining*. Cells stained for membrane proteins were washed and fixed using the fixation and permeabilization buffer (BioLegend) for 20 minutes at 4°C. This was followed by two washes with permeabilization buffer (1X) and intracellular labeling, carried out for 45 minutes at room temperature. Finally, cells were washed and resuspended in D-PBS for analysis via flow cytometry.

Protocol 3: *membrane* & *phosphorylated proteins immunostaining*. Membrane staining was done as in Protocol 1. Subsequently, cells were washed with PBS and fixed and permeabilized using fixation and permeabilization buffer (BioLegend) for 20 minutes at 4 °C. Following two washes, cells were labeled using unconjugated primary phosphorylated antibodies for 25 minutes at 4 °C. A wash was performed with permeabilization buffer, and secondary labeling was carried out using goat anti-mouse Dylight 488 FITC for 45 minutes. Finally, cells were washed and resuspended in D-PBS for analysis by flow cytometry.

### 2.12 *In-silico* database analysis

To examine the ligand–receptor (L–R) communication partners between activated CD8^+^T and NK cells in humans, we screened 2005 pairs established by the “CellChat” database (https://github.com/sqjin/CellChat), considering cell types. The expression defined as transcripts per million (TPM) data for humans was extracted from the Schmiedel database in the Human Protein Atlas (https://www.proteinatlas.org/). The selection of the communicating L–R pairs was stratified based on the criteria that each protein of the L–R pair expressed ≥ 10 or ≥ 2 TPM (**Supplementary Table S1**). In the case of either the ligand or the receptor form dimers, for example, [L–L]–[R] or [L]–[R–R], such as [ITGAL–ITGB2]–[CD226], we chose the pairs, given that each of all three proteins in the complex, [L–L]–[R] or [L]–[R–R], expressed ≥ 10 TPM.

### 2.13 Statistical analysis

Data were analyzed using the GraphPad Prism 10.2.3 (GraphPad Software Inc.) and presented as means ± SD. Comparisons between control and treatment groups were performed using a one-way analysis of variance (ANOVA) followed by Dunnett’s post-tests. Comparisons between the two groups were performed using two-tailed unpaired *t*-tests. Comparisons of survival curves obtained by Kaplan-Meïer plots were performed by the Mantel-Haenszel log-rank test. All statistical tests were two-sided, with *p*-values < 0.05 considered statistically significant.

## 3 Results

### 3.1 The pre-tumor adoptive T cell transfer in mice enables preemptive immunosurveillance against tumor development and antigen escape

**Figure 1:**
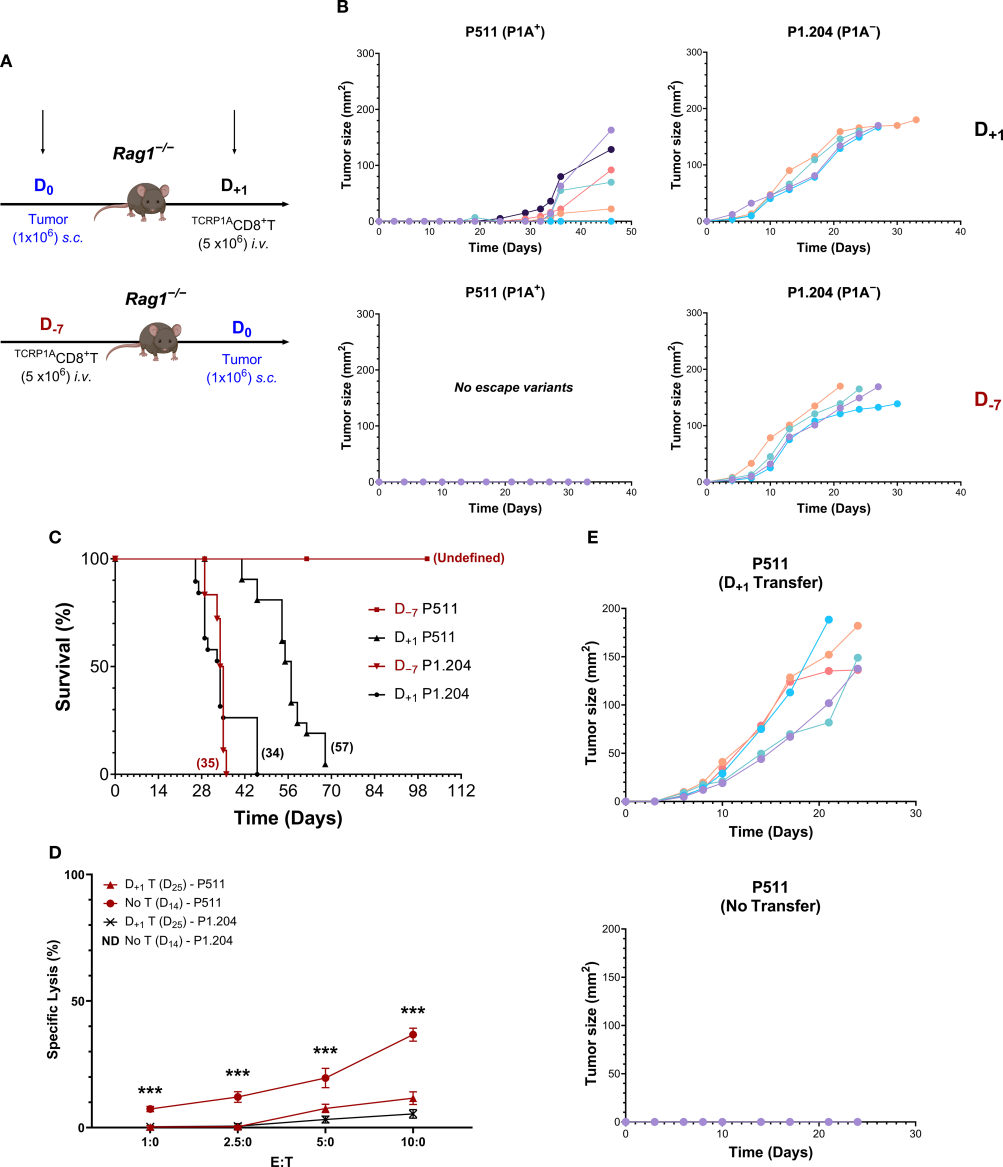
Pre-tumor adoptive T cell transfer enables immunosurveillance against tumor development and antigen escape in mice. **(A)** Designs of T cell transfer protocols are shown where *Rag1* ^−/−^B10.D2 mice were reconstituted with 5 x 10^6 TCRP1A^CD8^+^T cells 7 days (D_−7_) before or one day (D_+1_) after the subcutaneous injection of 1 x 10^6^ P1A^+^P511 or P1A^−^P1.204 tumor cells. **(B, C)** P511 or P1.204 tumor growth was monitored. Individual tumor growth kinetics **(B)** from one representative experiment, n = 5–7 mice per group, and Kaplan-Meier survival curves **(C)** from four experiments are presented. Numbers in parentheses depict median survival in days. n = 22 mice per group, *p*≤ 0.001 (two-sided log-rank test) for D_−7_ protocol in mice with P511 tumors compared with other groups. **(D)** ^TCRP1A^CD8^+^T cell cytolytic assay against P511 or P1.204 tumor cells growing out at day 25 following D_+1_ transfer of T cells in comparison with P511 tumor cells isolated at day 14 from *Rag1* ^−/−^B10.D2 mice without T cell transfer. Purified ^TCRP1A^CD8^+^T cells were stimulated *in vitro* for 3 days with syngeneic wt T-depleted splenic APCs loaded with the relevant cognate P1A_35–43_ peptide (LPYLGWLVF) and incubated with target cells for 5.5 h before analysis. ***, *p*≤ 0.001 (two-sided unpaired *t*-test) relative to other groups. **(E)** Tumor growth kinetics in ^TCRP1A^ *Rag1* ^−/−^B10.D2 mice of re-inoculation of outgrowing P511 tumor cells isolated at day 25 following D_+1_ transfer of ^TCRP1A^CD8^+^T cells in comparison with P511 cells harvested from *Rag1* ^−/−^B10.D2 mice without T cell transfer, n = 5.

To study the fate of tumor-specific CD8^+^T cells in mice, T cell adoptive transfer protocols were established with P511 tumors expressing a cancer-germline antigen P1A. In the pre-tumor homeostatic protocol (D_−7_), we reconstituted *Rag1* ^−/−^B10.D2 mice with 5x10^6^ CD8^+^T cells specific to H-2L*^d^*:1A_35-43_ (^TCRP1A^CD8^+^T) 7 days prior to subcutaneous tumor injection and compared with a post-tumor protocol (D_+1_), where the same number of ^TCRP1A^CD8^+^T cells were transferred one day after tumor injection (**Figure 1A**). In striking contrast to the outcomes of the D_+1_ protocol, the pre-tumor D_−7_^TCRP1A^CD8^+^T cell transfer protocol completely prevented growth of P511 tumor cells and provided long-term survival in all mice with no sign of tumor relapse (**Figures 1B, C**). Interestingly, the experimental conditions of the D_−7_ protocol eliminated the growth of tumor escape mutants compared with the D_+1_ protocol (**Figure 1B**). Furthermore, in the post-tumor D_+1_ protocol, most mice died of P511 tumors, although their median survival was prolonged relative to P1A-deficient P1.204 tumor-bearing mice injected with ^TCRP1A^CD8^+^T cells according to D_−7_ or D_+1_ protocol (median survival: 57 versus 34 or 35 days, respectively) (**Figure 1C**, **Supplementary Table S2**). Thus, there appears to be a control failure in the D_+1_ protocol to eliminate escape mutants developing at the tumor injection site, resulting in tumor relapse in mice after 3–4 weeks of tumor rejection, with most animals dead between 35 and 70 days (**Figure 1C**). In contrast, in the pre-tumor D_−7_ protocol, all the mice survived with no tumor escape (**Figures 1B, C**).

Examining the nature of P511 variants showed that tumor cells escaping on day 25 following D_+1_^TCRP1A^CD8^+^T cell transfer in *Rag1* ^−/−^B10.D2 mice were insensitive to ^TCRP1A^CD8^+^T cell-mediated specific lysis compared with tumor cells harvested from mice without T cell transfer (**Figure 1D**). Furthermore, on day 25, the tumor escape cells grew unrestricted in *^TCRP1A^Rag1* ^−/−^B10.D2 transgenic mice. In contrast, the tumors from mice without T cell transfer were rejected (**Figure 1E**). Since no downregulation of MHC class-I co-stimulatory molecules B7.1 or B7.2 or adhesion molecules LFA-1 or ICAM-1 was detected on escaping tumor cells (**Supplementary Figure S1**), this suggests an intact antigen presentation process but a loss of the expression of P1A tumor antigen.

### 3.2 Both direct and cross-antigen presentation effectively trigger the response of ^TCRP1A^CD8^+^T cells against P1A^+^ tumors

**Figure 2:**
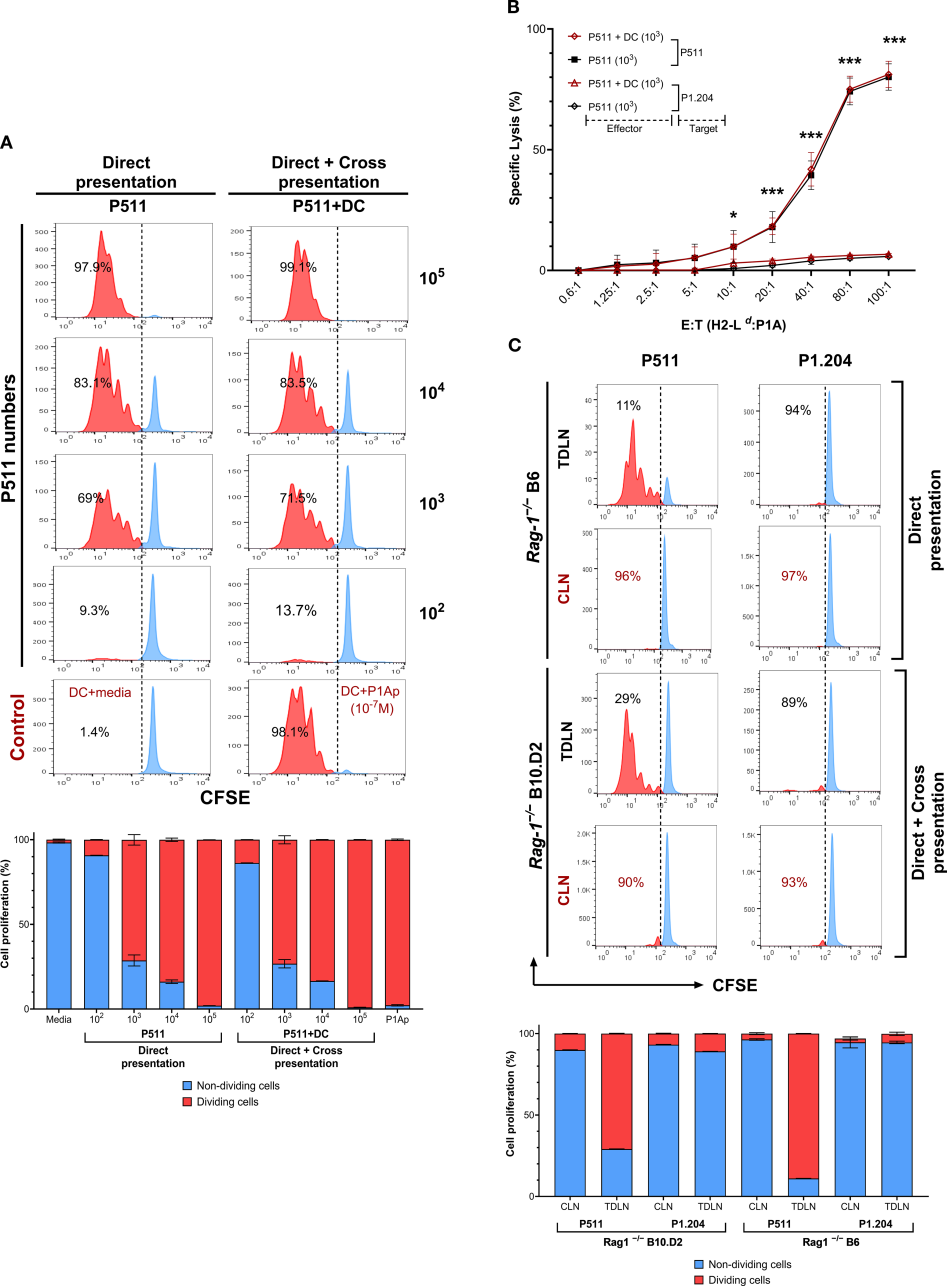
Direct P1A antigen presentation by tumor cells efficiently triggers CD8^+^T cell proliferation and elicits cytotoxic activity. **(A)** T cell proliferation is shown when naïve CFSE-labeled ^TCRP1A^CD8^+^T cells (0.5 x 10^6^) were cultured with indicated numbers of irradiated (20 Gy) tumor cells with or without 0.5 x 10^6^ syngeneic dendritic cells. Bar graphs: Quantitative summary of proliferation frequencies (%) per generation (red: proliferating; blue: non-proliferating cells) from histograms; Data represent mean ± SEM; n=2. The same number of dendritic cells loaded with or without 10^−7^ M P1A_35–43_ peptide (P1Ap) were used as controls. **(B)** Cytolytic activity against P1A^+^P511 and P1A^−^P1.204 targets are shown for CD8^+^T cells harvested from 1 x 10^3^ P511 co-culture with or without DC and compared to the P1A-deficit targets, n=3. *, *p* ≤ 0.05; ***, *p* ≤ 0.001. **(C)** The outcome of P1A tumor Ag presentation *in-vivo* is shown by T cell proliferation in tumor-draining lymph nodes (TDLN) and contralateral lymph nodes (CLN) on day two measured by CFSE divisions in P511 mismatched Rag1^−/−^B6 mice, with the possibility of only direct presentation of P1A by injected tumor cells or in P511 syngeneic Rag1^−/−^B10.D2 mice, where both direct as well as cross-presentation via host APCs could occur. Rag1^−/−^B6 and Rag1^−/−^B10.D2 mice were infused with CFSE-labeled ^TCRP1A^CD8^+^T cells (5 x 10^6^) 7 days after the subcutaneous injection of 1 x 10^6^ P1A^+^P511 or P1A^−^P1.204 tumor cells. Quantification of proliferation frequencies (%) in Rag1^-/-^ B6 mice and Rag1^−/−^B10.D2 is presented; n=2. Data are representative of at least two independent experiments. Values in the bar graphs are represented as mean ± SD.

We investigated the impact of direct or cross-presentation of tumor cell antigens on T cell activation by host antigen-presenting cells. Naïve ^TCRP1A^CD8^+^T cells were labeled with carboxyfluorescein diacetate succinimidyl ester (CFSE) dye and cultured with various concentrations of irradiated tumor cells in combination with or without syngeneic dendritic cells (DC). We found that direct P1A presentation by tumor cells was as efficient without DC as when cultured with DC in triggering T cell proliferation (**Figure 2A**, **Supplementary Table S3**). ^TCRP1A^CD8^+^T cells harvested from both conditions elicited identical levels of cytolytic activity against P1A^+^ targets (**Figure 2B**), suggesting no qualitative difference in the outcomes of direct or cross-presentation. To test this in the *in-vivo* condition, we injected P511 tumor cells in allogeneic *Rag1* ^−/−^B6 mice. In these mice, the absence of syngeneic APC allows only P1A direct presentation by tumor cells, whereas, in syngeneic *Rag1* ^−/−^B10.D2, both direct presentation by tumor cells and cross-presentation by host APC occurs. Interestingly, transferred ^TCRP1A^CD8^+^T cells showed an increase in the number of dividing cells with at least six divisions observed by day 2 in P1A^+^P511 tumor-draining lymph nodes (TDLN) of syngeneic or allogeneic mice compared with contralateral lymph nodes (CLN), even though the harvested T cell numbers were lower in *Rag1* ^−/−^B6 mice likely due to their alloreactive peripheral deletion by FasL-mediated apoptosis (**Figure 2C**, **Supplementary Table S4**). We tested the possibility of allogenic DC cross-dressing by trogocytosis. Our attempts at detecting L*^d^*:P1A peptide complexes on allogenic host DCs incubated with apoptotic or necrotic P1A^+^ tumors were negative. These results indicate that direct P1A antigen presentation by tumor cells without cross-presentation efficiently induces T cell proliferation and elicits tumor-specific CD8^+^T cell response.

### 3.3 ^TCRP1A^CD8^+^T lymphocytes detect and eliminate P1A-loss tumor variants through cooperative interactions with other immune cells

**Figure 3:**
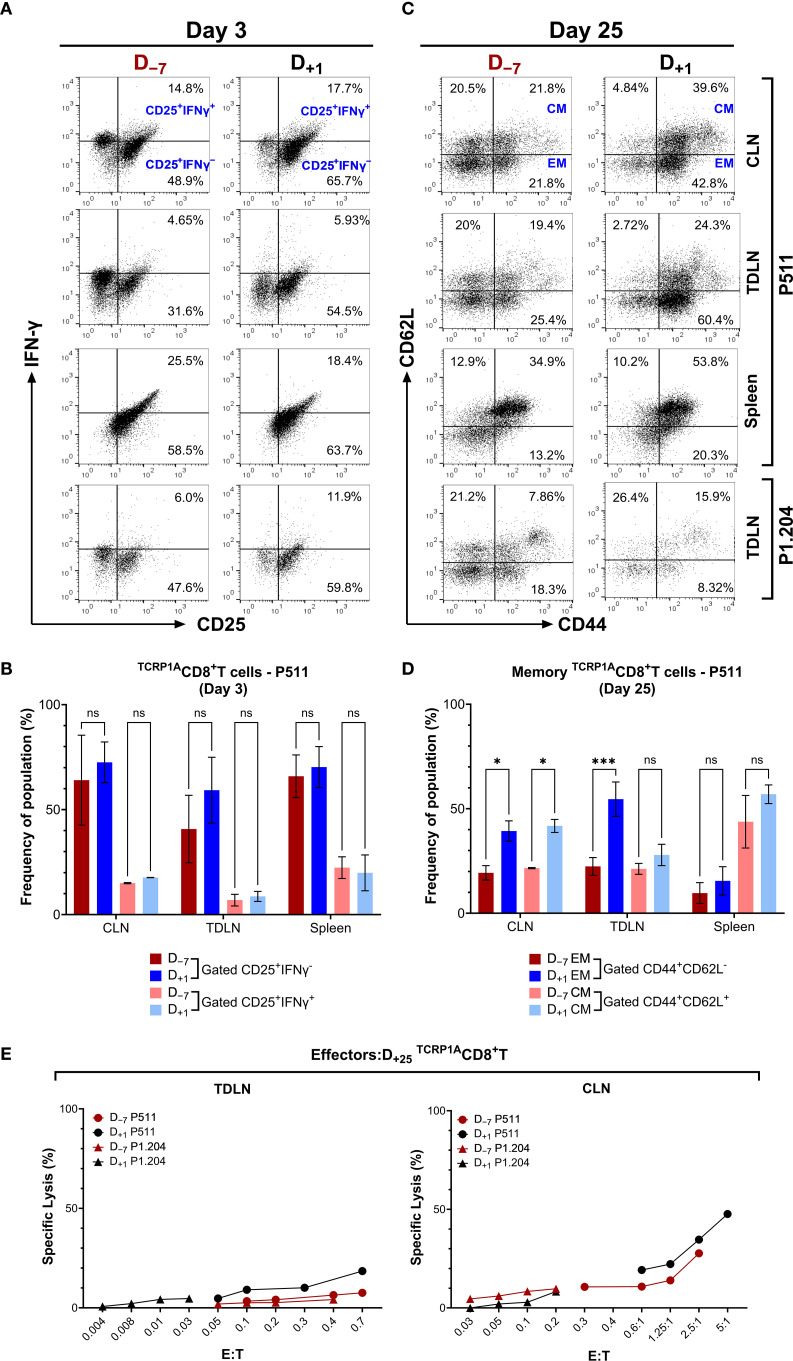
CD8^+^T cells following the pre– and post–tumor transfers exhibit comparable activation and memory effector function. **(A)** Representative flow cytometry plots showing co-expression of activation (CD25) and effector (IFNγ) markers on gated ^TCRP1A^CD8^+^ T cells in tumor-draining lymph nodes (TDLN) and contralateral lymph nodes (CLN) from Rag1^-/-^ B10.D2 mice, comparing D_–7_ and D_+1_ transfer protocols 3 days after tumor injection. Quadrants indicate percentages of positive populations. **(B)** Bar graph showing the population frequency (%) of CD25^+^ IFNγ^-^ and CD25^+^ IFNγ^+^ in CD8^+^T cells from CLN, TDL and spleen; Data represent mean ± SEM; n=2. **(C)** Corresponding analysis of CD44 and CD62L expression 25 days after tumor injection is shown on gated H2-L*d*:P1A tetramer^+^ T cells in the CLN, TDLN and spleens of Rag1^−/−^ B10.D2 mice that were infused with 5 x 10^6 TCRP1A^CD8^+^T cells 7 days (D_−7_) before or one day (D_+1_) after s.c. injection of 1 x 10^6^ P1A^+^P511 or P1A^−^P1.204 tumor cells. **(D)** Bar graph showing the population frequency (%) of CD44^+^ CD62L^-^ and CD44^+^ CD62L^+^ in CD8^+^T cells from CLN, TDLN and spleen; Data represent mean ± SEM; n=2. *, *p* ≤ 0.05; ***, *p* ≤ 0.001. **(E)** *Ex-vivo* cytolytic activity against P1A^+^ targets is shown for the CLN and TDLN cells harvested on day 25 post-tumor from P1A^+^P511 or P1A^−^P1.204 tumor-injected mice infused with D_+1_ or D_−7_^TCRP1A^CD8^+^T cell transfers. *, *p*≤ 0.05 (two-sided unpaired t-test) relative to CD8^+^T cells harvested from P1A^−^P1.204 tumor-bearing mice. Data are representative of two independent experiments.

We analyzed ^TCRP1A^CD8^+^T cell characteristics during pre- and post-transfer. Although examining the eliminated tumors in the pre-tumor D_−7_ protocol is not possible, we assessed the activation of adoptively transferred T cells. Three days after P511 tumor injection, H2-L*^d^*:P1A tetramer^+ TCRP1A^CD8^+^T cells from D_−7_ or D_+1_ protocol maintain expressed IL-2-receptor-α chain (CD25) and intracellular IFNγ at equivalent levels in tumor-draining lymph nodes (TDLN) and spleens, with insignificant expression observed in mice with P1A^−^P1.204 tumors (**Figures 3A, B**). Moreover, at day 3 post-transfer, ^TCRP1A^CD8^+^ T cells showed comparable frequencies and total counts in CLN and spleen between D_–7_ and D_+1_ groups, with a trend of reduction in frequency observed in TDLN in the D_+1_ group (**Supplementary Figure S2**). By day 25, proportions of effector memory CD44^high^CD62L^low^ cells (T_EM_) among ^TCRP1A^CD8^+^T cells in mice with D_+1_ protocol were ~2-fold higher in TDLN (*p* = 0.0007), contralateral lymph nodes (CLN; *p* = 0.0231) than the proportions observed in D_−7_ protocol (**Figures 3C, D**). The ^TCRP1A^CD8^+^T cells in the D_+1_ protocol also showed a predominantly higher percentage of CD44^high^CD62L^high^ phenotype characteristic of central memory cells (T_CM_) in the CLN (*p* = 0.0218) (**Figure 3C**). These memory-like populations were negligible in P1A^−^P1.204 tumors. Despite the higher memory phenotype in the D_+1_ group, both D_-7_ and D_+1_ protocols exhibited similar cytolytic activity against P1A^+^ targets at matched effector:target ratios (E:T) in TDLN and CLN (**Figure 3E**). Although ^TCRP1A+^CD8^+^T cells from the D_+1_ protocol exhibit higher proportions of memory phenotypes, both D_–7_ and D_+1_ groups show equivalent activation and cytotoxic function.

These results suggest that tumor elimination in the D_–7_ protocol is not solely dependent on T cell memory differentiation but may involve early clearance facilitated by optimal timing and homeostatic cooperative interactions with other immune cell populations. Conversely, sustained exposure to antigens in the D_+1_ group may not only promote the expansion of effector memory T cells but also elevate immune exhaustion. In addition, immune selection pressure from sustained effector activity in the D_+1_ protocol could lead to the emergence and survival of tumor variants exhibiting P1A antigen loss. Collectively, these findings underscore the collaborative role of non-T cell immune components in the complete clearance of tumors under the D_–7_ T cell transfer protocol.

### 3.4 Granzyme B–expressing NK cells, in concert with preemptive adoptive T cells, mediate the elimination of tumor escape variants

**Figure 4:**
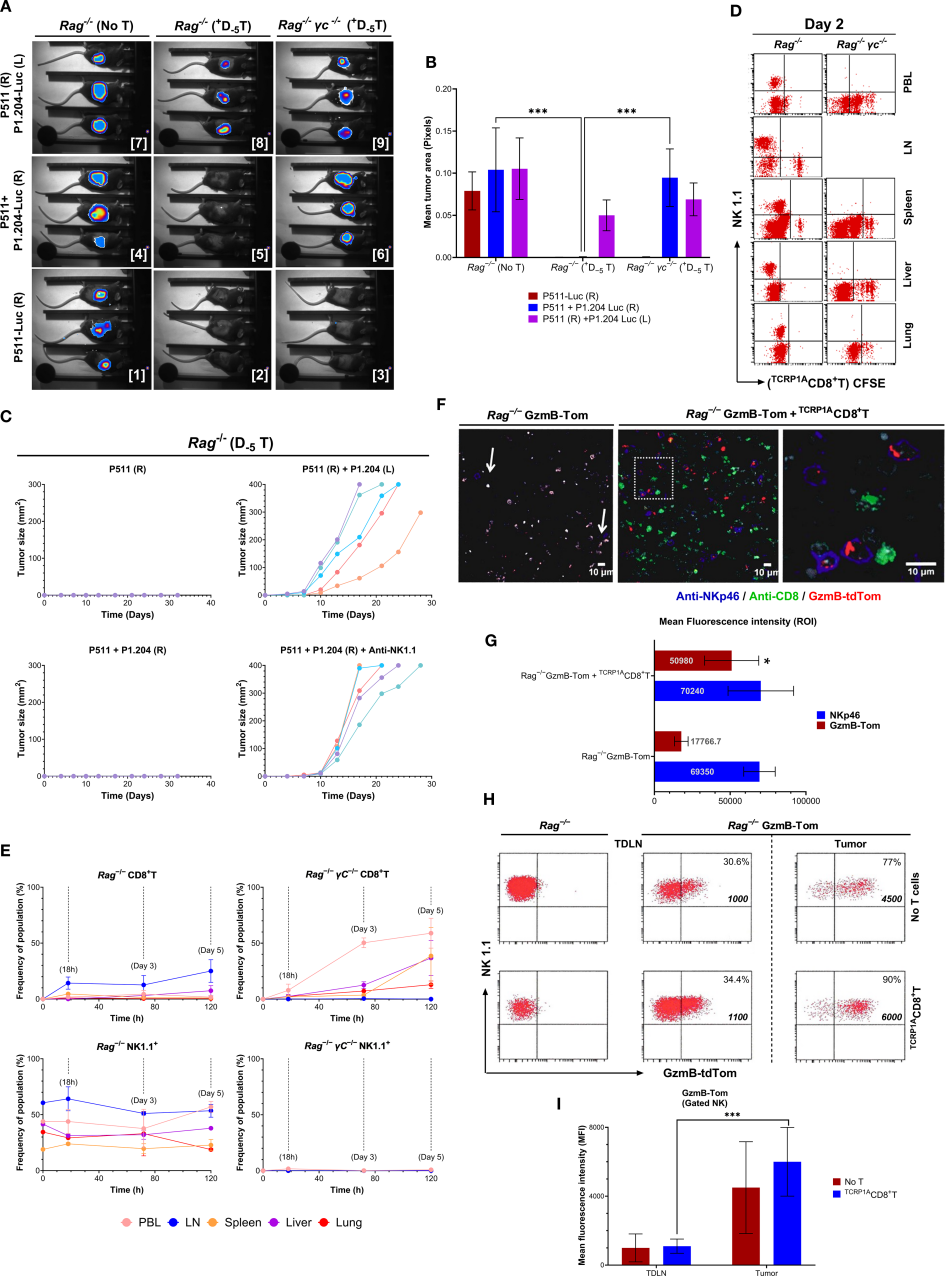
CD8^+^T cells enhance NK cell granzyme B expression to prevent tumor escape. **(A)** Bioluminescence imaging of tumor growth in *Rag1* ^−/−^B10.D2 or *Rag1* ^−/−^γc^−/−^B10.D2 mice injected s.c. with 1 x 10^6^ P1A^+^P511 (*right flank*, R) and 1 x 10^6^ P1A−P1.204-Luc (*left flank*, L), or a mixture of 1 x 10^6^ P1A^+^P511 and 1 x 10^6^ P1A^–^P1.204-Luc (R), or 2 x 10^6^ P1A^+^P511-Luc (R). All mice had received an i.v. transfer of 2 x 10^6 TCRP1A^CD8^+^T cells 5 days earlier (D_−5_). **(B)** Bar graph displaying the summary of luciferase bioluminescence intensities calculated by mean tumor area (pixels), reflecting tumor growth and cytotoxic response. ***, *p* ≤ 0.001. **(C)** Tumor growth curves of individual *Rag1* ^−/−^B10.D2 mice injected with the P511 and/or P1.204 tumor cells under four different flank conditions: 1. P511 (R): 1 x 10^6^ P511 cells injected into the right flank only. 2. P511 (R) + P1.204 (L): 1×10^6^ P511 cells on the right flank and 1 x 10^6^ P1.204 cells on the left flank. 3. P511 + P1.204 (R): 1 x 10^6^ P511 and 1 x 10^6^ P1.204 cells co−injected into the right flank. 4. P511 + P1.204 (R) + Anti−NK1.1: Co−injection as in (3), followed by NK cell depletion via anti−NK1.1 antibody treatment to confirm NK cell dependency. Each colored line represents the tumor volume (mm^2^) in a single mouse over time (days post−implantation). Tumor volumes were measured every 3–4 days. Data are representative of two independent experiments (n = 5 mice per group). **(D)** Transferred ^TCRP1A^CD8^+^T cell localization and proliferation as read by CFSE divisions on day two post transfer is shown in PBL, LN, spleen, liver, and lung. **(E)** Proportions of CD8^+^ and NK1.1^+^ cells are shown at 18h, and on day 3 and 5 post-transfer in the indicated tissues of *Rag1* ^−/−^B10.D2 or *Rag1* ^−/−^γc^−/−^B10.D2 mice; Data are mean ± SEM (n = 3–5 mice per group per timepoint). **(F)** Endogenous Tomato fluorescence corresponding to the GzmB-tdTom (GzmB) protein expression and co-staining with anti-NKp46 and anti-CD8 is mAb shown by confocal imaging of P1A^+^P511 tumor sections from *Rag1* ^−/−^B10.D2 or *Rag1* ^−/−^GzmB-TomB10.D2 mice five days after D_-7_ transfer with or without 3 x 10^6 TCRP1A^CD8^+^T cells injected i.v. A magnification of the insert is shown in the rightmost panel. **(G)** Mean fluorescence intensity (MFI) measured as region−of−interest (ROI) values for NKp46 (blue bars) and GzmB-tdTom (GzmB−Tom, red bars) in NK cells from *Rag1* ^−/−^GzmB-TomB10.D2 mice, either with or without CD8^+^T cell transfer. *, *p* ≤ 0.05. **(H)** Expression of GzmB-Tom and its mean fluorescence intensity in the NK1.1^+^ population is shown in the tumor-draining LN and P1A^+^P511 tumors from *Rag1* ^−/−^B10.D2 or *Rag1* ^−/−^GzmB-TomB10.D2 mice with or without ^TCRP1A^CD8^+^T cell transfer. Numbers in quadrants represent % positive cells and MFIs. **(I)** Bar graph showing mean fluorescence intensity (MFI) of the GzmB-tdTom in NK1.1^+^ NK cells isolated from the TDLN and tumor of *Rag1* ^−/−^GzmB-TomB10.D2 mice (n=3) either left untreated (No T; red bars) or receiving ^TCRP1A^CD8^+^T cell adoptive transfer (^TCRP1A^CD8^+^T; blue bars). Data are representative of three independent experiments. Values on the bar graphs are represented by the mean ± SD, ***p<0.001.

We further assessed whether the complete tumor elimination and containment of tumor escape by the preemptive T cell transfer were due to bystander effects following an induced adaptive response or other mechanisms mediated by alternative interactions with other immune cells. We established P1A^+^P511 and P1A^−^P1.204 tumors on the opposite flanks of the same *Rag1* ^−/−^ or *Rag1* ^−/−^ *γc* ^−/−^ mice. Based on kinetics established previously (15), transfer of ^TCRP1A^CD8^+^T cells on D_−5_ led to a rejection of only P1A^+^P511 tumors (*Images 2-3*), but not P1A^−^ tumors on the opposite flank, in both *Rag1* ^−/−^ and *Rag1* ^−/−^ *γc* ^−/−^ mice (*Images 8-9*, **Figure 4A**). However, when P511 and P1.204 tumors were established at the same site, D_−5_ transferred ^TCRP1A^CD8^+^T cells rejected P1A^−^P1.204 tumors in *Rag1* ^−/−^ but not *Rag1* ^−/−^ *γc* ^−/−^ mice (*Images 5-6*, **Figures 4A, B**). Furthermore, P511 tumors alone (*top left*, **Figure 4C**) or co-injected with the P1.204 variant on the right flank (*bottom left*) exhibited no measurable growth over time, indicating an effective immune response. Notably, depletion of NK cells by administration of anti-NK1.1 antibody (*bottom right*, **Figure 4C**) led to enhanced growth of P1.204 tumors, supporting the role of NK cells in controlling antigen escape.

Moreover, transferred ^TCRP1A^CD8^+^T cells were traced in the LN and spleen of *Rag1* ^−/−^ mice on day 2 with scant proliferation, while they showed vigorous proliferation in the PBL, spleen, liver, and lungs of *Rag1* ^−/−^ *γc* ^−/−^ mice (**Figure 4D**). Five days later, ^TCRP1A^CD8^+^T cells represented a major proportion in the PBLs (65%), spleen (40%), liver (40%) and lungs (10%) of *Rag1* ^−/−^ *γc* ^−/−^ mice (*top right*), with no NK cells detected (*bottom right*, **Figure 4E**). Contrastingly, transferred T cells in *Rag1* ^−/−^ mice represented < 3% in the PBL or spleen and ~25% in LN on the fifth day (*top left*), with a significant proportion of NK cells in all tissues (> 50% in LN and PBL, *bottom left*, **Figure 4E**, and **Supplementary Figure S3**), Despite an extensive NK cell distribution, *Rag1* ^−/−^ mice did not reject mixed P1A^+^ and P1A^−^ tumors without the transferred T cells. Furthermore, NK-deficient *Rag1* ^−/−^γc^−/−^ mice transferred with ^TCRP1A^CD8^+^T cells did not reject P1A^−^P1.204 tumors. Mixed tumor cells were rejected only in D_−5_^TCRP1A^CD8^+^T cell-transferred NK cell-replete *Rag1* ^−/−^ mice. Collectively, these results illustrate that neither CD8^+^T cells nor NK cells, in isolation, possess the capacity to eradicate antigen-deficient tumors. Instead, their synergistic interaction is imperative for the successful rejection of tumor antigen–escape variants, underscoring the interplay of T–NK cell crosstalk in the immune surveillance of heterogeneous tumor cells.

Hence, we imaged the cellular interaction and granzyme B expression of endogenous NK cells *in situ* with transferred ^TCRP1A^CD8^+^T cells in P1A^+^P511 tumors of *Rag1* ^−/−^ and *Rag1* ^−/−^ GzmB-Tom mice (**Figure 4F**). Indeed, NK cells co-localized with transferred ^TCRP1A^CD8^+^T cells (**Figure 4F**) and demonstrated an endogenous tomato fluorescence corresponding to GzmB significantly higher compared with NK cells without ^TCRP1A^CD8^+^T interaction (*p* = 0.0235) (**Figure 4G**), in tumors compared to tumor-draining LN (*p *< 0.0001) (**Figures 4H, I**). Thus, without relying on bystander effects of ^TCRP1A^CD8^+^T cell cytotoxicity to eliminate antigen-deficient tumor variants, NK cells with augmented expression of GzmB in presence of tumor-infiltrating CD8^+^T cells prevent tumor establishment and antigen escape. Moreover, the increase in GzmB expression observed in the *in vivo* model (**Figures 4F–I**) may not directly correlate with NKG2D ligand levels expressed in the CD8^+^T cells or NK cells (**Supplementary Figure S10**) but rather reflect a broader NKG2D-agnostic activation profile of NK cells resulting from immune interactions within the tumor microenvironment. These findings suggest that the communication and interaction between CD8^+^T cells and NK cells play a pivotal role in eliminating emerging immune escape tumor variants.

### 3.5 Antitumoral CD8^+^T−NK cell crosstalk relies on pseudopodial intercellular membrane fragment exchange

**Figure 5:**
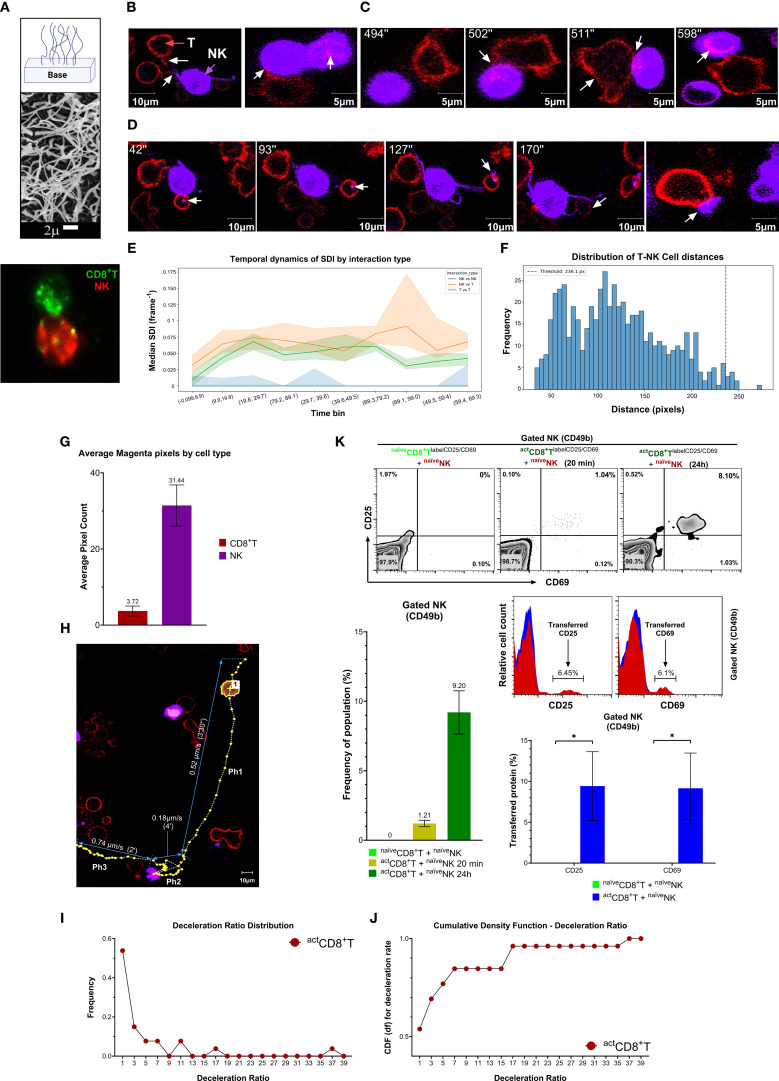
Pseudopodial membrane tunneling nanotube-like structures mediate physical interactions between CD8^+^T and NK cells. **(A)** Diagram and electron micrograph of 3D silica nanofiber carpet made of ε-polycaprolactone nanofibers ranging from 200 nm in diameter at the base to 100 nm at the tip and 20 to 50 μm in length, similar in size to reticular fibers such as collagen III. The fibers were spaced every 2 μm and tethered to the base substrate, creating a gradient of stiffness through the thickness of nanofibers analogous to the basement membrane to detect an optimal condition to visualize the migration and support of lymphocytes. **(B)** Laser scanning confocal microscopy images of transitory membranous nanotubes (white arrows) formed by ^naïve^NK (purple) or activated ^act^CD8^+^T (red) cells during their interaction. The pseudopodial contact and transfer of membrane fragment are visible from ^act^CD8^+^T to the membrane of ^naïve^NK. **(C)** The cellular kinetics of membrane invagination, protrusion, and transfer marked by white arrows are shown in a time-lapse imaging from ^act^CD8^+^T cell (red) into ^naïve^NK (purple). **(D)** Membrane transfer (white arrows) is shown from ^naïve^NK (purple) to ^naïve^CD8^+^T cells (red) by time-lapse confocal imaging. The rightmost panel shows the transferred membrane on CD8^+^T cell. **(E)** Line plot showing median Speed-Distance Index (y−axis; units of Shannon index per cell) across successive time bins (x−axis; binned by hours post−interaction as indicated) for three distinct NK−NK, NK−T and T−T intercellular interaction modalities. Each colored line denotes the median SDI for one interaction type, with the shaded envelope representing the interquartile range. **(F)** Histogram displaying the frequency of measured distances (in pixels) between CD8^+^T cell and NK cell centroids across all analyzed fields (bin width = 10 pixels). The vertical red dashed line at 250 pixels denotes the predefined proximity threshold used to classify cell pairs as “close” (≤ 250 pixels) versus “distant” (> 250 pixels). **(G)** Bar graph representing average pixel counts of transferred membrane fragments (magenta) quantified in CD8^+^ T and NK cells. **(H)** Representative image showing the migration trajectory of a CD8^+^T cell interacting with NK cells across three behavioral phases: Ph1 (pre-contact), Ph2 (contact), and Ph3 (post-contact). The yellow line indicates the cell track; instantaneous velocities (μm/s) are annotated. The significant drop in T cell velocity during Ph2 illustrates interaction-induced deceleration. **(I, J)** Deceleration ratios (Ph2/Ph1) plotted for individual CD8^+^T cells (n = 20). Each red dot represents a single cell. The Y-axis shows the velocity deceleration ratio [Velocity during contact (Ph2)/Velocity before contact (Ph1)], while the X-axis indicates the cell index number (not a measurement). More than 70% of cells exhibit a deceleration ratio ≤ 0.2, indicating a ≥ 5-fold motility reduction during NK cell contact. **(K)** Membrane CD25 and CD69 expression on ^act^CD8^+^T and ^naïve^NK cells. The upper panel shows CD25 and CD69 expression zebra dot plots on gated unlabeled ^naïve^NK cells after the co-culture with ^naïve^CD8^+^T (left plot) or with activated ^act^CD8^+^T labeled with fluorochrome-conjugated antibodies to CD25 and CD69 for 20 min (middle) and 24 h (right). Lower panels show corresponding histograms and bar graphs for the transferred CD25 and CD69 expression detected on the membrane of unlabeled ^naïve^NK cells with or without interaction with ^act^CD8^+^T. Data are representative of at least three independent experiments. Values are mean ± SD, *p = 0.03.

To investigate the physical and functional dynamics of synaptic interactions between CD8^+^T cells and NK cells, and their implications for antitumor immunosurveillance, we analyzed cell-cell contact and motility behaviors using confocal laser-scanning microscopy. Imaging lymphocyte interactions *in vitro* is technically challenging due to their high intrinsic motility. To overcome this, we developed a three-dimensional (3D) reticular nanofiber silica carpet of fiber density ranging from 25 fibers per 100 μm² in standard sections to 4 fibers per 100 μm² in the 5 × 5 mold region with larger fiber diameters. This mimicked the density and topography of collagen III-rich extracellular matrix (ECM) environments in loose connective tissues, including lymphoid tissues (23–26). Under these 3D conditions, we observed direct interactions and membrane fragment exchange between CD8^+^T (*Green*) and NK cells (*red*) (**Figure 5A**). However, due to the limited optical accessibility of the dense matrix, we complemented this system with 2D substrate conditions using fibronectin, which allowed high-resolution imaging and quantification of intercellular contact events.

In these experiments, NK cells were labeled with lipophilic long-chain dialkyl carbocyanine tracer, DiD (*purple*), and CD8^+^T cells with cholesterol-binding laurdan (*red*). Labeled CD8^+^T and NK cells seeded onto fibronectin pre-coated plates demonstrated round shapes and either moved randomly or stayed immobilized. However, upon approaching each other (direct contact), NK cells also formed intercellular membrane nanotubes following prominent pseudopodial invaginations and protrusions (**Figure 5B**, white arrows; **Supplementary Video S1**), allowing the transfer of membrane fragments from CD8^+^T to NK apparent in a time-lapse imaging (**Figure 5C**, white arrows; **Supplementary Video S2**) and *vice versa* from NK to T cells (**Figure 5D**, white arrows; **Supplementary Video S2**).

To quantify the dynamics of CD8^+^ TNK cell colocalization, we segmented time-lapse videos into 100 frames and applied spatiotemporal modeling. Cell boundaries were defined using their respective fluorescent profiles, and intercellular proximity and centroid shifts were tracked over time (**Supplementary Figure S4**). We used the Speed–Distance Index to calculate interaction intensity, which is defined as a function of cell velocity and intercellular distance. CD8^+^T–NK cellular pairs exhibited significantly higher SDI values relative to other interactions, indicating directional migration and stable contact, characteristic of immune synapse formation (**Figure 5E**, **Supplementary Table S5**). Furthermore, a bimodal distribution of colocalization frequency by distance (**Figure 5F**) suggested the directed attraction of CD8^+^T cells toward NK cells. We then quantified magenta fluorescence within each cell type, revealing measurable signal exchange between CD8^+^T and NK cells, further supporting membrane transfer during interaction (**Figure 5G**).

Additionally, to assess the kinetics of interactions between CD8^+^T cells and NK cells, we tracked individual CD8^+^T cell motility across three distinct kinetic phases: before contact (*Ph1*), during contact (*Ph2*), and after separation (*Ph3*) (**Figure 5H**). Cells decelerated upon engagement with NK cells, followed by acceleration after disengagement. This kinetic pattern was quantified using a deceleration ratio (Ph2/Ph1), and cumulative analysis revealed that over 70% of CD8^+^T cells reduced speed by ≥ 5-fold during contact (**Figures 5I, J**), consistent with stable immune synapse formation for the sustained synaptic engagement and signaling.

Finally, to explore the functional consequences of this interaction, we assessed whether activating receptors or molecules could be transferred between cells. Labeled naïve or activated CD8^+^T cells stained with fluorochrome-conjugated CD25 and CD69 antibodies were co-incubated with the naïve unlabeled NK cells. Flow cytometry analysis showed a small fraction (~1%) of CD69^+^CD25^+^ NK cells at 20 minutes, which increased to 6–8% by 24 hours. In contrast, no such transfer was observed when ^naïve^CD8^+^T cells were used (**Figure 5K**). Thus, CD8^+^T−NK cell crosstalk occurs through pseudopodial tunneling nanotube-like structures, enabling trogocytosis and the directed transfer of activating membrane receptor fragments from ^act^CD8^+^T to ^naïve^NK cells. Although these results do not take into account any transcriptional contributions of CD25 or CD69 molecules, they demonstrate that antitumor CD8^+^T–NK interactions engage dynamic, polarized pseudopodial nanotube-like structures that facilitate bidirectional activating membrane receptor exchange through trogocytosis. This exchange not only reflects physical contact but also supports the transfer of functional molecules critical for the cellular activation machinery.

### 3.6 Receptor–ligand interactions during CD8^+^T−NK cell crosstalk are essential for regulating effector mechanisms and promoting memory cell formation

**Figure 6:**
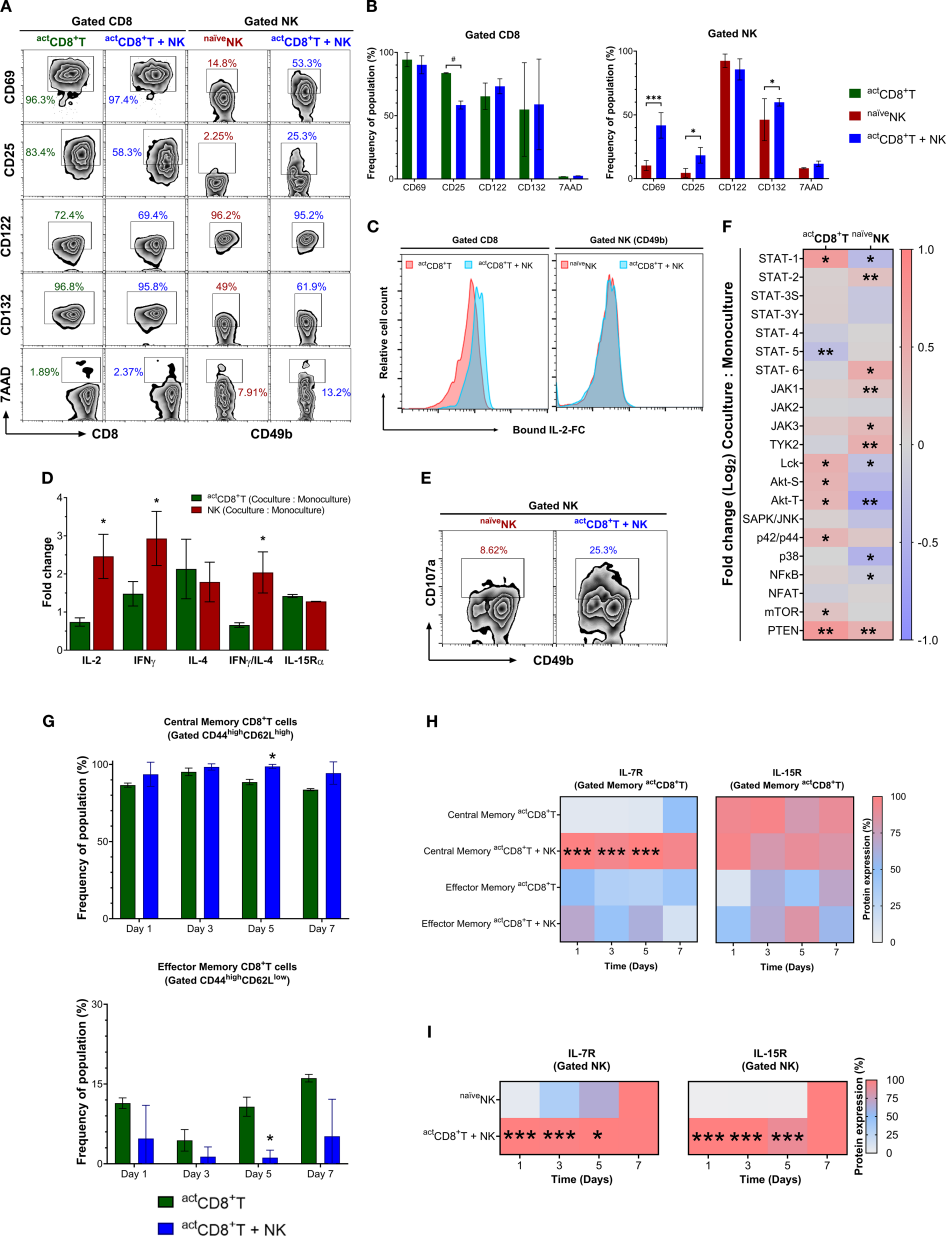
Activated CD8^+^T cells and naïve NK cells engage in reciprocal crosstalk. **(A)** Expression of CD69, CD25, CD122, and CD132 molecules is shown from gated activated CD8^+^T cells cultured alone (^act^CD8^+^T) or together with naïve NK cells (^act^CD8^+^T + ^naïve^NK) or from gated naïve NK cells cultured alone (^naïve^NK) or together with activated CD8^+^T cells (^act^CD8^+^T + ^naïve^NK) for 24 h **(B)** Quantitative flow cytometric assessment of surface activation markers (CD69, CD25, CD122, CD132) and viability (7AAD) on gated activated CD8^+^ T cells (^act^CD8^+^T, green), naïve NK cells (^naïve^NK, red), and cells from co-culture (^act^CD8^+^T + NK, blue). Data is shown as mean ± SEM. Values mean ± SD. #, *p* = 0.08; *, *p* ≤ 0.05; ***, *p* ≤ 0.001. **(C)** Bound intracellular IL-2-Fc staining in gated ^act^CD8^+^T or NK cells cultured alone (red) or together (blue) for 24 h **(D)** Fold−change analysis of intracellular IL−2, IFN−γ, IL−4, the IFN−γ/IL−4 ratio, and IL−15Rα chain measured in activated CD8^+^T cells (green bars) and NK cells (red bars) following 24 h co-culture versus monoculture. Data are expressed as mean ± SEM from three independent flow experiments. *, *p* ≤ 0.05. **(E)** Expression analysis of CD107a by NK cells cultured alone or with ^act^CD8^+^T cells for 36 h **(F)** The heatmap graph shows fold changes in the proportion of phospho-forms of indicated signaling proteins (pX) normalized to their total content (X) in CD8^+^T cells or NK cells co-cultured with ^naïve^NK or ^act^CD8^+^T cells. The ratio [pX, Co-culture: total X, Co-culture] was normalized to the ratio [pX, Monoculture: total X, Monoculture] obtained for individual (CD8^+^T or NK) cell cultures as per the formula [pX, Co-culture: total X, Co-culture]/[pX, Monoculture: total X, Monoculture]. *, *p* ≤ 0.05; **, *p* ≤ 0.01. **(G)** Bar graphs of CD44 versus CD62L on gated central (CD44^+^CD62L^+^) and effector (CD44^+^CD62L^–^) memory CD8^+^T cells in co-culture compared with monoculture. *, *p* ≤ 0.05. **(H)** Heatmap graph showing the dynamics of surface expression changes of IL-7R and IL-15R in the central (CD44^+^CD62L^+^) and effector (CD44^+^CD62L^–^) ^act^CD8^+^T cells co-cultured with ^naïve^NK cell or alone using an *in vitro* D_–7_ protocol. ***, *p* ≤ 0.001. **(I)** Heatmap graph showing the dynamics of changes in IL-7R and IL-15R expression in ^naïve^NK (CD49b^+^CD11b^+^) co-cultured with ^act^CD8^+^T cells or alone. Numbers depict % positive cells. Data are representative of at least three independent experiments. Values are mean ± SD, *, *p* ≤ 0.05; ***, *p* ≤ 0.001.

To gain a deeper understanding of the collaborative mechanisms between CD8^+^T and NK cells against tumor variants, we conducted an analysis of receptor–ligand engagement, cytokines and downstream molecules that may play a role in their activation and effector function. We established co-cultures consisting of equal numbers of activated CD69^high^CD25^high^ (≥ 90%) CD8^+^T cells (^act^CD8^+^T, 0.5 x 10^6^) and naïve CD49b^+^ (DX5^+^) NK cells (^naïve^NK, 0.5 x 10^6^) in fibronectin-precoated plates (**Figure 6A**).

**Figure 7:**
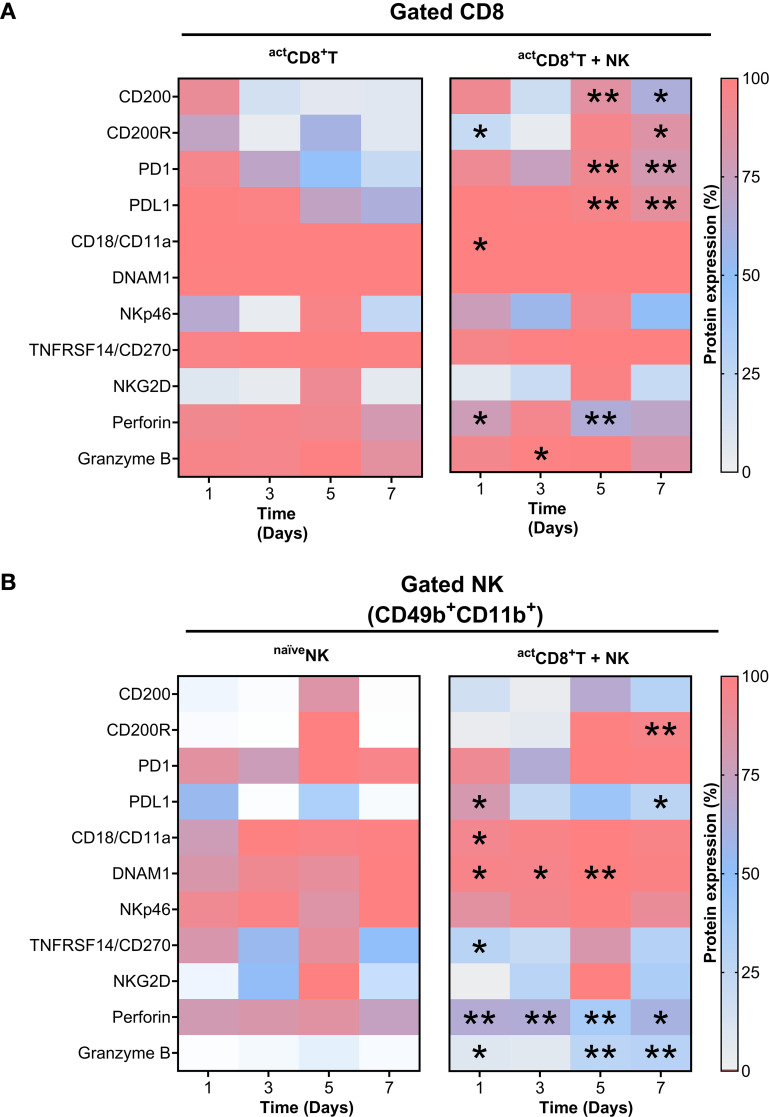
Time-dependent upregulation of key protein network molecules through CD8^+^T–NK cell interactions. Heatmaps showing relative changes of protein expression in CD8^+^ T cells **(A)** and NK cells **(B)** under monoculture (left) and co-culture (right) (*in vitro* D_–7_ protocol) conditions at indicated time points. Asterisks denote statistically significant differences compared to monoculture controls; *, *p* ≤ 0.05; **, *p* ≤ 0.001.

After 24 h co-culture, ^act^CD8^+^T dampened the inducible IL-2-receptor-α (CD25) but not the constitutive β (CD122) and γ (CD132) chains or CD69 expression. On the other hand, NK cells, upon *co-culture*, upregulated CD25, CD132, and CD69 without affecting CD122 compared with NK cells in *monoculture*. CD8^+^T cells and NK cells in monocultures or co-cultures showed no increased cell death according to 7-AAD measurements (**Figure 6B**). Moreover, despite a decrease in CD25 but not in CD122 and CD132 chains, CD8^+^T cells showed an increased capture of recombinant IL-2/Fc chimera protein. In contrast, NK cells did not show any change in the captured IL-2/Fc protein (**Figure 6C**). Fold-change analysis of intracellular cytokine expression in co-culture versus monoculture conditions, following the gating strategy shown in **Supplementary Figure S5**, demonstrated higher frequencies of NK cells (*red bars*) with increased relative expression of IL-2, IFN-γ, and IL-4, as well as a higher IFN-γ/IL-4 ratio when co-cultured with activated CD8^+^T cells (**Figure 6D**). Notably, IL-15Rα surface expression increased, albeit comparable between both cell types following co-culture (**Figure 6D**), indicating a shift in cytokine levels. This suggests that CD8^+^T–NK crosstalk promotes a pro-inflammatory, functionally cooperative response. An upregulation of IL-15Rα and IL-2Rβ expression on NK cells after co culture, while markers indicative of synapse formation (e.g., LFA-1, DNAM-1) reached a plateau (**Figure 7**), suggests that IL-15, which is critical for NK cell survival, proliferation, and cytotoxicity, along with IL-2, which is secreted by activated CD8^+^T cells, play essential roles in driving the effector functionality of NK cells. Indeed, an elevated IFNγ:IL-4 ratio in co-cultured NK cells (**Figure 6D**) and increased degranulation molecule CD107a observed after 36 h co-culture in gated NK cells support an NK cell effector profile (**Figure 6E**).

To elucidate the signaling dynamics following interactions between CD8^+^T cells and NK cells and to correlate these dynamics with their effector mechanisms, we performed a flow array to analyze the total and phosphorylated levels of intracellular kinases, phosphatases, and transcriptional factors covering cytokine and immune receptor signaling pathways (**Supplementary Figure S6**). Following co-culture, NK cells displayed increased phosphorylation of JAK1, JAK3, and TYK2 kinases, along with STAT2 and STAT6 transcription factors. Additionally, they showed a significant reduction in LCK and p38 kinases, as well as STAT1 and NFκB. These components mediate the receptor signaling of interleukins IL-4 and IL-15 (**Figure 6F**, *right side*). Conversely, ^act^CD8^+^T cells displayed decreased levels of phospho-STAT5, which regulates the IL-2 receptor signaling pathway. Interestingly, we observed increased levels of phosphorylated STAT1 without any noticeable changes in the JAK1–3 or TYK2 kinases in CD8^+^T cells. Despite this, several components downstream of TCR stimulation, including LCK, PTEN, AKT mTOR, and p42/p44 MAPK (but not p38), demonstrated a significant increase in phosphorylation (**Figure 6F**, *left side*).

We also evaluated the implications of CD8^+^T–NK cell crosstalk in the generation of CD8^+^T memory-like cell subtypes during 7 days of co-culture (*in vitro* D_–7_ protocol) (**Supplementary Figure S7**). We observed an increased proportion of central memory-like CD8^+^T cell phenotype (CD62L^high^CD44^high^), a subpopulation phenotype observed in co-culture compared to monoculture, which may indicate that CD8^+^T–NK cells crosstalk is responsible for the generation of effector responses and central memory T-cell differentiation. In contrast, a lower proportion of the effector memory-like CD8^+^T cell phenotype (CD62L^low^CD44^high^) was observed during co-culture (**Figure 6G**). Data show that central memory-like ^act^CD8^+^T cells co-cultured with NK cells exhibit a higher expression of the IL-7 receptor (IL7R) (**Figures 6H, I**), suggesting a role of IL-7 in the survival and improved response of memory T lymphocytes and NK cells. Furthermore, we observed a marked increase in IL-15R expression in the co-cultured NK cells (**Figure 6I**). These findings underscore the critical role of CD8^+^T and NK cell interactions in achieving an adequate immune response, which is fundamental for tumor clearance and preventing the escape of tumor variants.

### 3.7 Human immune network database analysis identified ligand–receptor pair interactions underlying CD8^+^T cell−NK cell crosstalk

Our previous in-silico analysis showed the potential role of the ligand–receptor (L-R) interactions for a comprehensive understanding of the intrinsic mechanisms of communication between CD8^+^T and NK cells (27, 28). Building on this foundation, we expanded the analysis by leveraging recent open-source databases. Specifically, we utilized the CellChat database to analyze 2005 L–R interaction pairs in humans. Focusing on activated CD8^+^T and NK cells, we filtered this dataset using expression profiles from the Schmiedel dataset (Human Protein Atlas, 2022), which includes data for over 12,200 unique genes (29). Applying a conservative threshold of ≥ 10 transcripts per million (TPM), we identified 40 L–R pairs; using a more inclusive threshold of ≥ 2 TPM, this increased to 105 L–R pairs (**Supplementary Table S1**).

Detailed analysis of these L–R pairs revealed nine in the first set (40 L–R with ≥ 10 TPM) and additional 16 proteins with a wide range of potential interactions in the second set (105 L–R with ≥ 2 TPM), which are directly related to activation, proliferation, migration, effector and regulatory functions, and memory generation of CD8^+^T cells and NK cells (**Table 1**). Furthermore, we identified 27 potential interactions specifically relevant to immune functions (**Supplementary Table S1**).

**Table 1:** Key ligand–receptor (L–R) pairs mediating CD8^+^T–NK cell crosstalk and associated immune functions.

Expression Threshold		≥ 2 TPM*		≥ 10 TPM		Potential Immune Function
≥ 2 TPM	≥ 10 TPM	Ligand	Receptor	Ligand	Receptor	Role
YES	YES	TNFSF10	TNFRSF10A	TNFSF10	TNFRSF10A	Activation
YES	YES	TNF	TNFRSF1A	TNF	TNFRSF1A	Activation
YES		SEMA7A	PLXNC1			Memory generation
YES	YES	SEMA4D	CD72	SEMA4D	CD72	Effector function
YES		SELPLG	SELP			Migration/Infiltration
YES	YES	SELPLG	SELL	SELPLG	SELL	Migration/Infiltration
YES		CD274	PDCD1			Regulatory function
YES		GZMA	F2R			Regulatory function
YES	YES	DLL1	NOTCH1	DLL1	NOTCH1	Effector function
YES	YES	TNFSF14	TNFRSF14	TNFSF14	TNFRSF14	Activation
YES	YES	ITGAL/ITGB2	CD226	ITGAL/ITGB2	CD226	Activation/Effector function
YES	YES	FASLG	FAS	FASLG	FAS	Regulatory function
YES		EFNA1	EPHA4			Regulatory function
YES		CD99	PILRA			Activation
YES	YES	CD6	ALCAM	CD6	ALCAM	Effector function
YES		BAG6	NCR3			Effector function
16^#^	9					

*Transcripts per million: L–R pairs categorized into two expression thresholds: ≥ 2 TPM (left columns) and ≥ 10 TPM (right columns). For each threshold, ligands and their corresponding receptors are listed alongside their functional roles, including activation, proliferation, memory generation, migration/infiltration, effector functions, and regulatory functions in CD8^+^T and NK cells.

^#^The numbers at the bottom of the table indicate the total counts of interactions in each TPM category.

This analysis demonstrates that CD8^+^T cells and NK cells have a strong potential for crosstalk through a wide range of coordinated interactions between the ligands on one cell type and the receptors on the other. From the two sets of L–R pairs, we selected those with significant immunological relevance, specifically the ones associated with the effector function of CD8^+^T cells and NK cells. We focused on a subset of L–R pairs known to be involved in effector functions, including CD200–CD200R1, PD-L1–PD-1, and CD18/CD11a–DNAM-1. These were further evaluated experimentally using an *in vitro* D_–7_ co-culture model, integrating temporal expression data (**Supplementary Figures S8**, **S9**, **Supplementary Table S6**). This analysis revealed dynamic and coordinated expression patterns indicative of functionally directed immune crosstalk. Notably, CD200 and its inhibitory receptor CD200R1 showed a marked increase in expression on both CD8^+^T cells and NK cells by day 5, which was sustained through day 7, suggesting a potential bidirectional regulatory interaction (**Figure 7**). Furthermore, CD8^+^T cells consistently expressed high levels of CD18/CD11a, DNAM-1, and PD-1, with expression maintained across all time points (**Figure 7A**). In parallel, NK cells exhibited elevated CD18/CD11a expression and a significant upregulation of DNAM-1, peaking at day 5, while PD-L1 remained low throughout the co-culture (**Figure 7B**). Dynamic changes sustained in the CD200–CD200R1 expression upon co culture indicates CD200 expression on T cells signaling through the increased CD200R1 on NK cells, thereby regulating NK cell function.

To further explore cytotoxic regulation, we analyzed the expression of additional activation and effector molecules, including TNFRSF14, NKG2D, and NKp46, and molecules related to the cytotoxic activity of NK cells and CD8^+^T cells. Importantly, NK cells demonstrated a progressive increase in granzyme B expression, reaching its highest levels between days 5 and 7. (**Figure 7B**). These findings align with *in vivo* observations showing increased granzyme B expression (**Figures 4G–I**). In addition, the co-culture of CD8^+^T and NK cells showed a trend of decreased surface expression of NKG2D ligands, specifically MULT-1 and H60 on gated CD8^+^T or NK cells (**Supplementary Figure S10**, **Supplementary Table S6**). These data suggest that the chosen L–R pairs may play a crucial role in interaction networks during CD8^+^T cell–NK cell crosstalk, facilitating effective communication between these cells in tumor-related scenarios, involving the presence of new antigen variants.

Together, these data highlight a temporally regulated pattern of receptor–ligand expression that likely modulates activation, effector function, and immune regulation during CD8^+^T cell–NK cell interaction. Functional validation of these receptor–ligand pairs will be essential to delineate their specific roles in orchestrating this crosstalk, particularly within the context of antitumor immunity.

## 4 Discussion

Dynamic cellular interactions within immune networks establish intricate feedback loops, finely regulating tissue-specific immune responses. In this study, we demonstrate that CD8^+^ T cells and NK cells engage in critical functional interactions mediated by diverse cellular and molecular mechanisms, significantly contributing to antitumor immunity and limiting the development of immune–escape variants in solid tumors. We showed that early homeostatic interactions of NK cells with CD8^+^T cells result in a significant enhancement of their cytotoxic capacities. When NK cells come into contact with CD8^+^T cells, they acquire an activated phenotype characterized by elevated endogenous granzyme B and perforin levels.

Our live-cell confocal microscopy provided direct evidence that interactions between CD8^+^T and NK cells are mediated through membranous contacts involving pseudopodial structures. In this sense, the pseudopodial nanotubes employed during some processes, such as trogocytosis, commonly documented in immune cells (30) are typically associated with facilitating bidirectional membrane fragment exchange in immune cells, allowing the transfer of molecules such as CD25 and CD69. Quantitative analysis also revealed a significant reduction in cellular motility (up to 97.5%) during these interactions, indicative of stable immune synapse formation. This contact-dependent immobilization is likely necessary for efficient membrane exchange. Indeed, immune interactions rely on diverse molecular structures, including connexin-43 gap junctions (31), LFA-1, lipid rafts (32), and specialized membrane channels (33, 34) that collectively enhance the stability and functional integrity of these intercellular synapses. Thin pseudopodial protrusions forming during T–NK contact—mediating bidirectional membrane transfer and showing stable SDI/deceleration—are consistent with intercellular membrane tunneling nanotubes.

Moreover, these synapse-like interactions are facilitated by a diverse array of molecular mechanisms, including immune and cytokine receptors. Our earlier *in-silico* analysis demonstrated that CD8^+^T and NK cells display distinct sets and quantities of co-stimulatory and co-inhibitory molecules, mimicking an interface between professional APCs (such as DC or B cells) and CD8^+^T cells (27). Our *in-silico* analysis using the Schmeichel database revealed additional ligand–receptor pairs with a high potential to be dynamic regulators of the CD8^+^T–NK cell crosstalk. Considering the number of verified and putative interacting molecular pairs between CD8^+^T and NK cells, these lymphocytes appear to form immune synapse-like contacts. In addition, during the CD8^+^T–NK crosstalk observed in our *in-vitro* D_–7_ protocol, a simulation of the *in-vivo* D_–7_ protocol, we observed a notable increase in the expression of IL-7 and IL-15 receptors (IL-7R and IL-15R, respectively). These IL-7/IL-7R and IL-15/IL-15R axes play critical roles in the development, homeostasis, and maintenance of cells of lymphoid origin, particularly memory T lymphocytes and NK cells (35, 36).

Furthermore, these cytokines are essential for enhancing the antitumor response and have demonstrated efficacy as antitumor agents, leading to tumor regression and the inhibition of lung tumor metastasis (37, 38). The increase in receptor expression may enhance T and NK cell responses to proliferative signals, leading to a more efficient response to tumors, particularly during the emergence of new immune escape variants, as evidenced in the D_–7_ protocol. Moreover, the increase in the expression of IL7R and IL15R in NK cells was also observed, and this was associated with heightened proliferation and enhanced effector capabilities to combat tumors (39, 40). These findings suggest that the interaction between CD8^+^T cells and NK cells is a critical factor in the antitumor immune response, with cytokine receptors playing a crucial role in this process. Additionally, we observed an increase in CD18/CD11a and DNAM-1 expression during T–NK cell crosstalk. This observation is significant because, although these molecules do not bind to each other, both tend to be recruited. This recruitment may cooperatively mediate specific processes within the immune synapse in T and NK cells by stabilizing adhesion and amplifying signals from activation molecules that can be transferred during crosstalk (41, 42). Such mechanisms may also facilitate an increase in the transfer of membrane fragments and enhance bidirectional communication. Conversely, the increase in the expression of ligand–receptor pairs related to inhibitory control, including both PD-1–PD-L1 and CD200–CD200R, suggests a state of activation in CD8^+^T cells and NK cells, as these receptors are upregulated following activation as a negative feedback mechanism for maintaining homeostasis (43–46).

We also demonstrated that NK cells conditioned by CD8^+^T cells exhibit increased secretion of IFNγ and IL-2. This cytokine increase suggests the remodeling of intracellular signaling pathways, as confirmed by the increased phosphorylation of JAK1, TYK2, STAT2, and STAT5, downstream targets of interferon receptor signaling, contributing to NK cell stimulation (47). In addition, increased phosphorylation of JAK1, JAK3, and STAT6, associated with IL-4 receptor signaling in NK cells, suggest the development of a mixed regulatory NK cell phenotype. As part of their immunoregulatory function, NK cells have been shown to dampen IL-2 signaling in CD8^+^T cells *via* down-regulation of surface IL-2Rα expression, IL-2-mediated intracellular pathways (STAT5 phosphorylation), and IL-2 synthesis along with elevated IL-2 endocytosis in these T cells (48). We tested whether NK cells capture IL-2 from the environment, thus limiting its availability to activated T cells. However, NK cells did not show any alterations in IL-2 capture. Being a high-affinity chain for the IL-2 receptor, CD25 is subject to fast internalization after its engagement but rapidly recycles to the cellular membrane after endocytosis (49). We showed a positive transfer of CD25 from CD8^+^T cells to NK cells during crosstalk. Based on this evidence, we propose that the regulatory effect of NK cells is not directly linked to competition for IL-2 but rather may be directly related to mechanisms of acceleration of CD25 downregulation/recycling. Another explanation is that CD25 molecules are directly transferred through the exchange of membrane fragments via pseudopodial nanotubes during crosstalk.

Another regulatory mechanism of NK cells is favoring the Th2-type cytokine profile in CD8^+^T cells. Although both cytokines (IFNγ and IL-4) are elevated in CD8^+^T cells after interaction with NK cells, IL-4 synthesis is increased to a higher extent, thus lowering the IFNγ : IL-4 ratio. CD8^+^T cells from co-culture with NK cells also exhibit downregulated IL-2 and STAT5-mediated signaling necessary for IFNγ expression in immune cells (50). Sequestration of CD8^+^T cells from IL-2 and STAT5-mediated signaling may restrict primary immune response and help preserve the pool of memory CD8^+^T cells for a secondary response (51); thus, it supports the observation that NK cell-conditioned CD8^+^T cells upregulate STAT1 phosphorylation, which is known to promote memory cell formation (52).

We also showed that phosphorylation of STAT1, a transcriptional mediator of IFNγ signaling, was downregulated in NK cells, suggesting an active intrinsic autoregulation of the IFNγ signaling pathway between CD8^+^T and NK cells during the crosstalk or competition for this cytokine. In our previous study, we demonstrated that the expression of engaged IFNγ-R1 on TCRP1A CD8^+^T cells was higher in P511 TILs compared to the tumor-draining lymph nodes (LN), whereas the expression of IFNγ-R1 remained unchanged on NK cells (14). IFNγ is known to upregulate the expression of TNF superfamily members on subsets of NK cells (53). Furthermore, tumor-infiltrating activated CD8^+^T cells may provide cytokines such as IL-2 and chemokines CCL3 and CCL4, which can activate NK cells (14). Thus, drawing from our murine model and the data obtained from the co-culture of CD8^+^T cells and NK cells, it is evident that cytokines derived from CD8^+^T cells within the crosstalk enhance the functionality of NK cells. This enhancement promotes a mixed phenotype in NK cells, which not only increases their antitumor efficacy but also moderates the activity of CD8^+^T cells and promotes the generation of central memory subsets.

**Figure 8:**
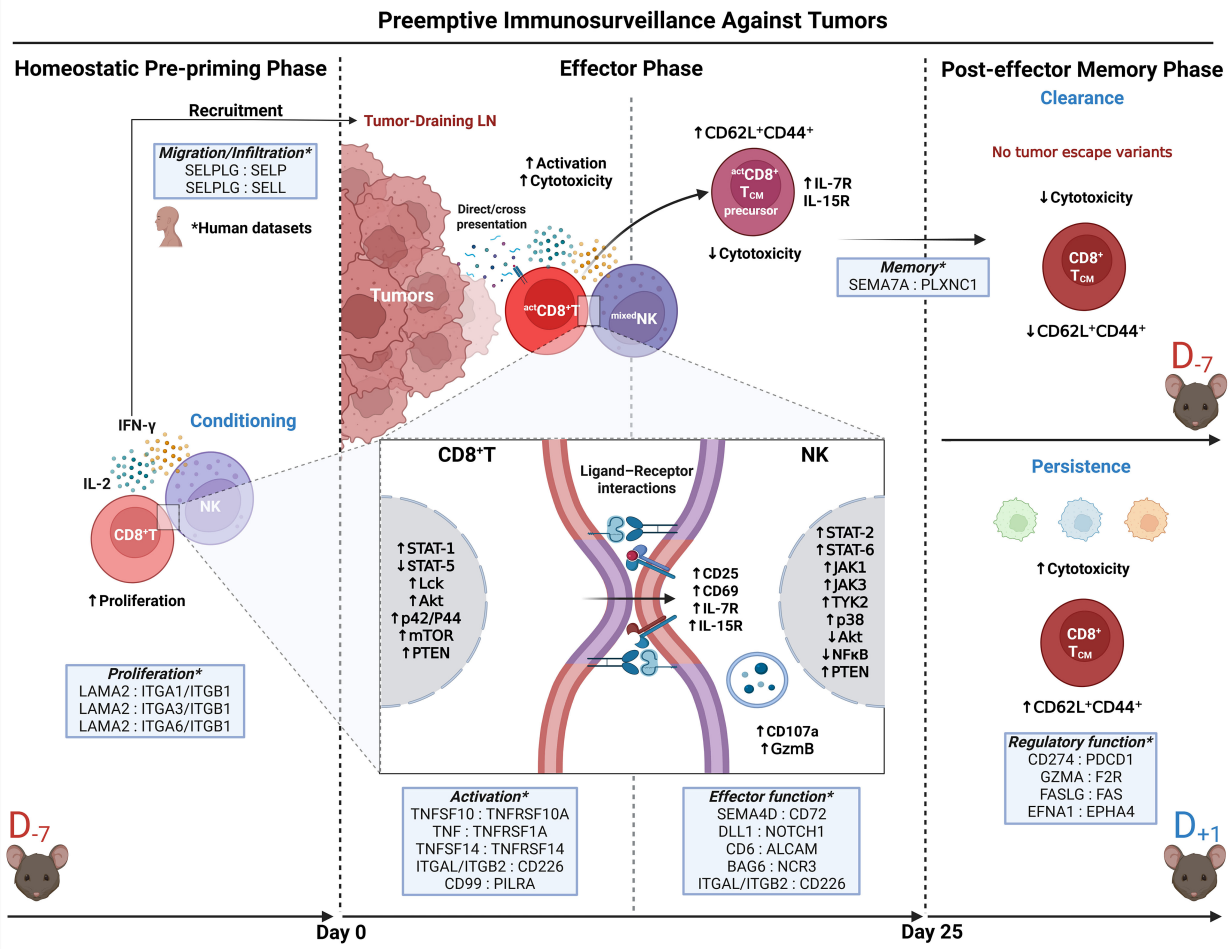
Three-phase model of preemptive immunosurveillance against tumors. Early crosstalk between CD8^+^ T cells and NK cells orchestrates three sequential phases—homeostatic pre-priming, effector, and post-effector memory—that mount an appropriate response against emerging tumor antigen–escape variants. In the *homeostatic pre-priming phase*, early reciprocal interactions, established by pre-tumor adoptive T cell transfer, provide tonic conditioning, trigger activation signals, and induce IFN-γ–dependent chemokines, driving spontaneous NK cell activation and recruitment to tumors and tumor-draining lymph nodes. During the *effector phase*, following direct antigen presentation and/or cross-presentation by APCs, sustained CD8^+^T–NK ligand–receptor engagement elicits robust CD8^+^T cell cytotoxicity and differentiation toward central memory (T_CM_) precursors, in concert with NK effector activity (e.g., CD107a, granzyme B degranulation). In the *post-effector memory phase*, the pre-tumor adoptive T cell transfer exhibits reduced T_CM_ persistence as antigen–escape tumor variants are efficiently cleared. By contrast, post-tumor T cell transfer faces ongoing antigenic evolution under antigen-specific T cell pressure, sustaining T_CM_ persistence. Moreover, regulatory mechanisms mediated partly by NK cells balance cytotoxic activity, effector persistence, and T cell memory formation. Thus, CD8^+^ T–NK crosstalk is essential for preemptive immunosurveillance, restraining tumor antigen escape. Analyses of human datasets (blue boxes) corroborate ligand–receptor pairs involved in CD8^+^ T–NK physical interactions, underscoring their clinical relevance.

Based on our findings, we propose a three-phase model for tumor preemptive immunosurveillance mediated by CD8^+^T and NK cell crosstalk (**Figure 8**). Within the context of lymphocyte response, during the *homeostatic pre-priming phase* of immune cell reconstitution and proliferation dominated by IL-2 and IFNγ cytokine milieu simulated in the D_–7_ T cell transfer protocol, potential conditioning signals (e.g., CD25, CD69, IL-7R, IL-15R) may be constantly exchanged to activate NK cells. Also, other molecules, including IFNγ signaling-related chemokines provided through this interaction, may facilitate the recruitment of T cells and NK cells to sites where tumors subsequently lodge (e.g., tumors and TDLN) (54). Once the tumor-conditioned microenvironment is established, during the *effector phase*, the sustained ligand–receptor interaction between CD8^+^T and NK cells, along with the intrinsic mechanisms to control the tumor, such as direct antigen presentation or possible cross-presentation by APCs, allows the rapid development of a coordinated response to tumors before the appearance of new tumor antigen–escape variants (55, 56). In addition, it was noted that NK cells can develop both the intrinsic effector mechanism against tumor cells and the regulatory function supporting differentiation of central memory formation by CD8^+^T lymphocytes. Lymphocyte memory subsets thus generated are characterized by a remarkable ability for proliferation and differentiation and are regarded as a transitional state between naïve T cells and T_EM_ at the molecular level during the dynamic differentiation processes (57).

The outcome of this synaptic interaction is demonstrated in the *post-effector memory phase* by observing the response of CD8^+^T lymphocytes on day 25 in both T cell transfer protocols. On the one hand, in the D_–7_ protocol, a decrease in T_CM_ and T_EM_ lymphocyte populations is observed compared to the D_+1_ protocol due to the absence of tumor antigenic variants. On the other hand, the D_+1_ protocol represents a scenario where the constant generation of antigenic variants with the lack of requisite ligands could be a limiting factor for the subsequent response launched by new memory CD8^+^T lymphocyte clones. These conclusions align with other studies related to the analysis of CD8^+^T cell response using mathematical modeling, which explains that during the expansion and formation of memory CD8^+^T cells, memory cell clones with fewer divisions can create more effective secondary responses to new tumor antigen variants (58).

In summary, the physical ligand–receptor interplay between CD8^+^T and NK cells mediated in part through pseudopodial intercellular membrane nanotubes plays a pivotal role in restraining tumor progression and countering immune evasion, underscoring its significance in preemptive tumor immunosurveillance. Our ongoing studies indicate that this intercellular dialogue is regulated by mitochondrial calcium dynamics (Uzhachenko, Ochoa, Shanker et al., unpublished). The findings uncover a new mechanistic axis of immunosurveillance, offering a foundation for next-generation therapies that can preempt and intercept tumor antigen–escape variants.

## Data Availability

The raw data supporting the conclusions of this article will be made available by the authors, without undue reservation.
