# Supplementary material for: CD8+ T–NK cell crosstalk establishes preemptive immunosurveillance to eliminate antigen–escape tumors
Source: Front Immunol. 2025 Sep 22;16:1593913. doi: 10.3389/fimmu.2025.1593913 (PMC12497863; doi:10.3389/fimmu.2025.1593913)
Supplement: Supplementary file 1 [file DataSheet1.docx]

Supplementary Material

#

# SUPPLEMENTARY FIGURES


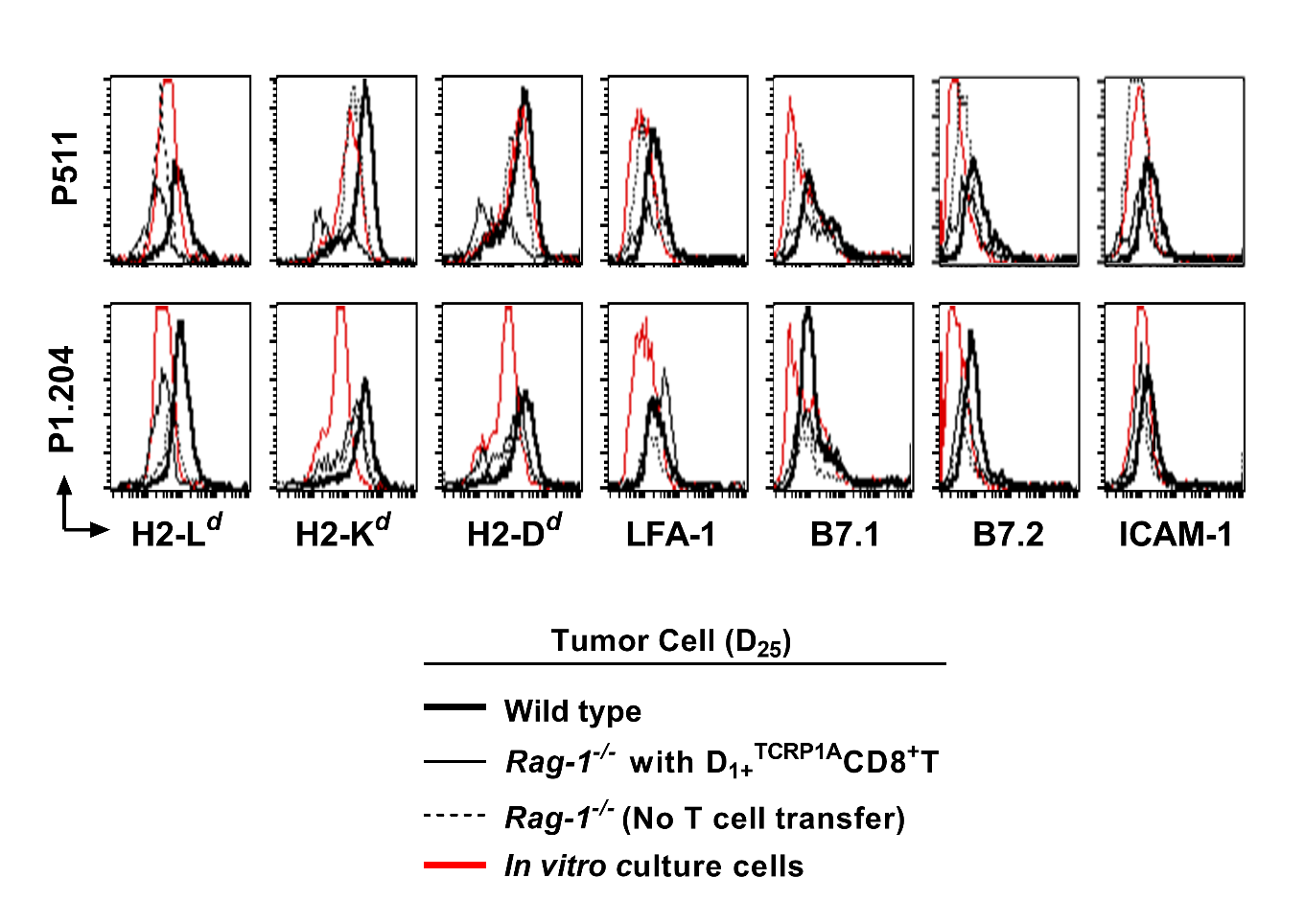


**SUPPLEMENTARY FIGURE S1.** The expression overlay of indicated molecules is shown on P511 or P1.204 tumor cells isolated on day 25 from wild-type, *Rag1*^−/−^B10.D2 with D_+1_ transfer of TCRP1CD8^+^T cells and *Rag1*^−/−^B10.D2 mice without T cell transfer. The expression was compared to the baseline expression on tumor cells in culture (red line). The analysis is shown from one representative experiment with five mice in each group.

**SUPPLEMENTARY FIGURE S2.** Bar graphs showing population frequencies and total numbers of TCRP1A⁺ CD8⁺T cells in the contralateral lymph node (CLN), tumor‑draining lymph node (TDLN), and spleen derived from the D_‑7_ (red bars) and D_+1_ (blue bars) protocols.

**SUPPLEMENTARY FIGURE S3.** Bar graphs showing frequencies of CD8⁺ (top) and NK1.1⁺ (bottom) cells in peripheral blood lymphocytes (PBL), lymph node (LN), spleen, liver, and lung on Day 5 in *Rag1*⁻^/^⁻ (blue bars) versus *Rag1*⁻^/^⁻ *γc*⁻^/^⁻ (red bars) mice.


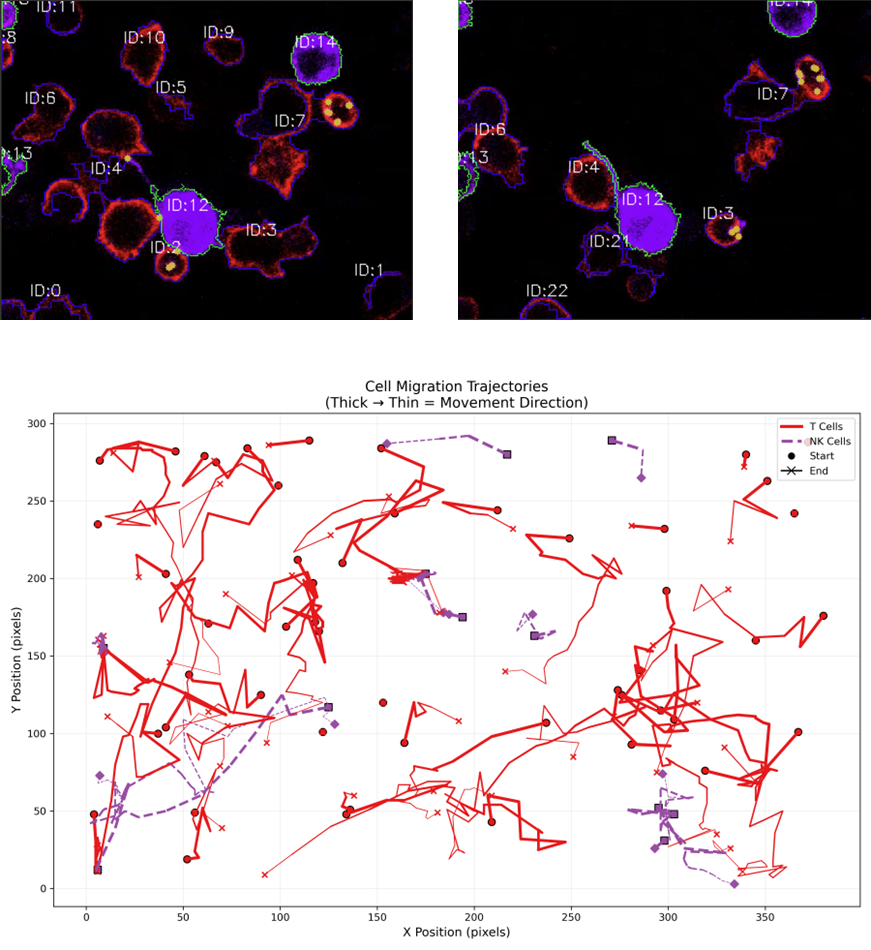


**SUPPLEMENTARY FIGURE S4.** Top: Representative confocal micrographs (two sequential frames) showing CD8⁺T cells (Red) and NK cells (Purple) within the imaging field. Overlaid cell outlines are colored blue for CD8⁺T cells, green for NK cells, and magenta merge pixels inside cells by yellow; numeric labels denote individual cell IDs. Bottom: Cell migration trajectories for the same population over the recording period. Solid red lines trace CD8⁺T cell paths; dashed purple lines trace NK cell paths. Line thickness tapers from thick to thin to indicate movement direction. Circles (●) mark trajectory start points; crosses (×) mark endpoints. Axes show X and Y positions in pixels.


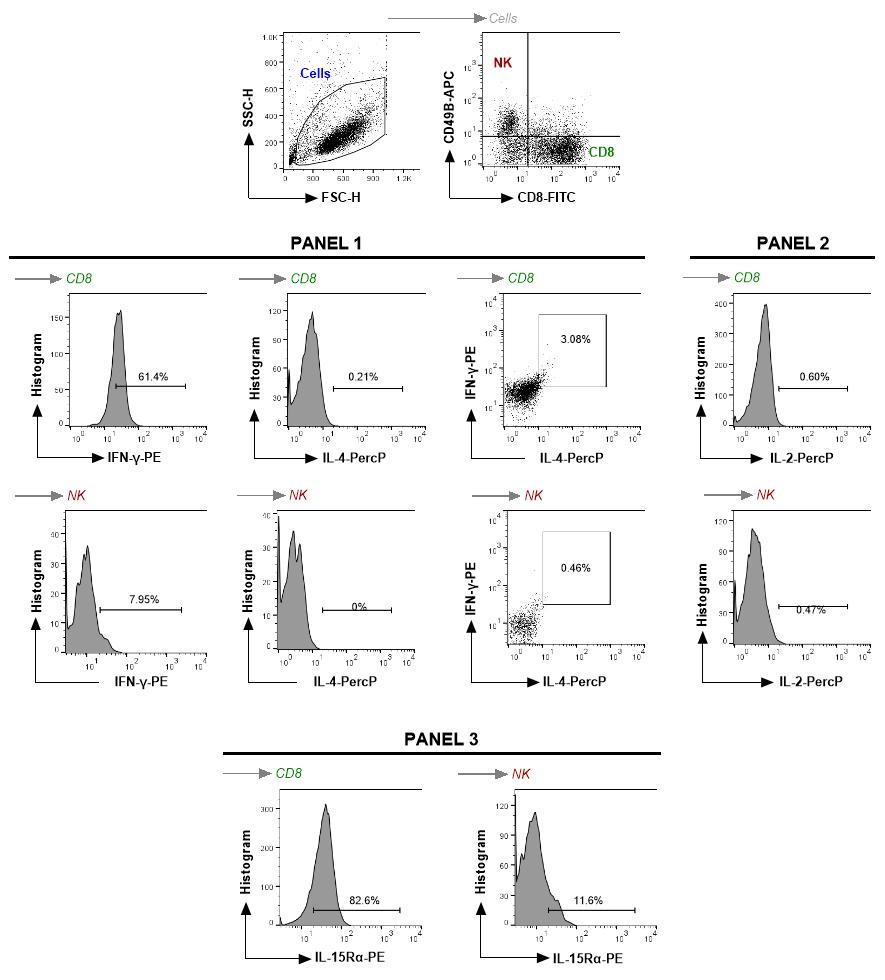


**SUPPLEMENTARY FIGURE S5.** Flow cytometry analysis strategy to assess intracellular cytokine and IL‑15Rα expression in CD8⁺T and NK cells using three antibody panels. Lymphocytes were first gated by FSC/SSC and discriminated into CD8⁺ and NK subsets; Panel 1 measured intracellular IFN‑γ, Panel 2 measured intracellular TNF‑α, and Panel 3 measured surface IL‑15Rα, with representative dot plots and histograms shown for each population.


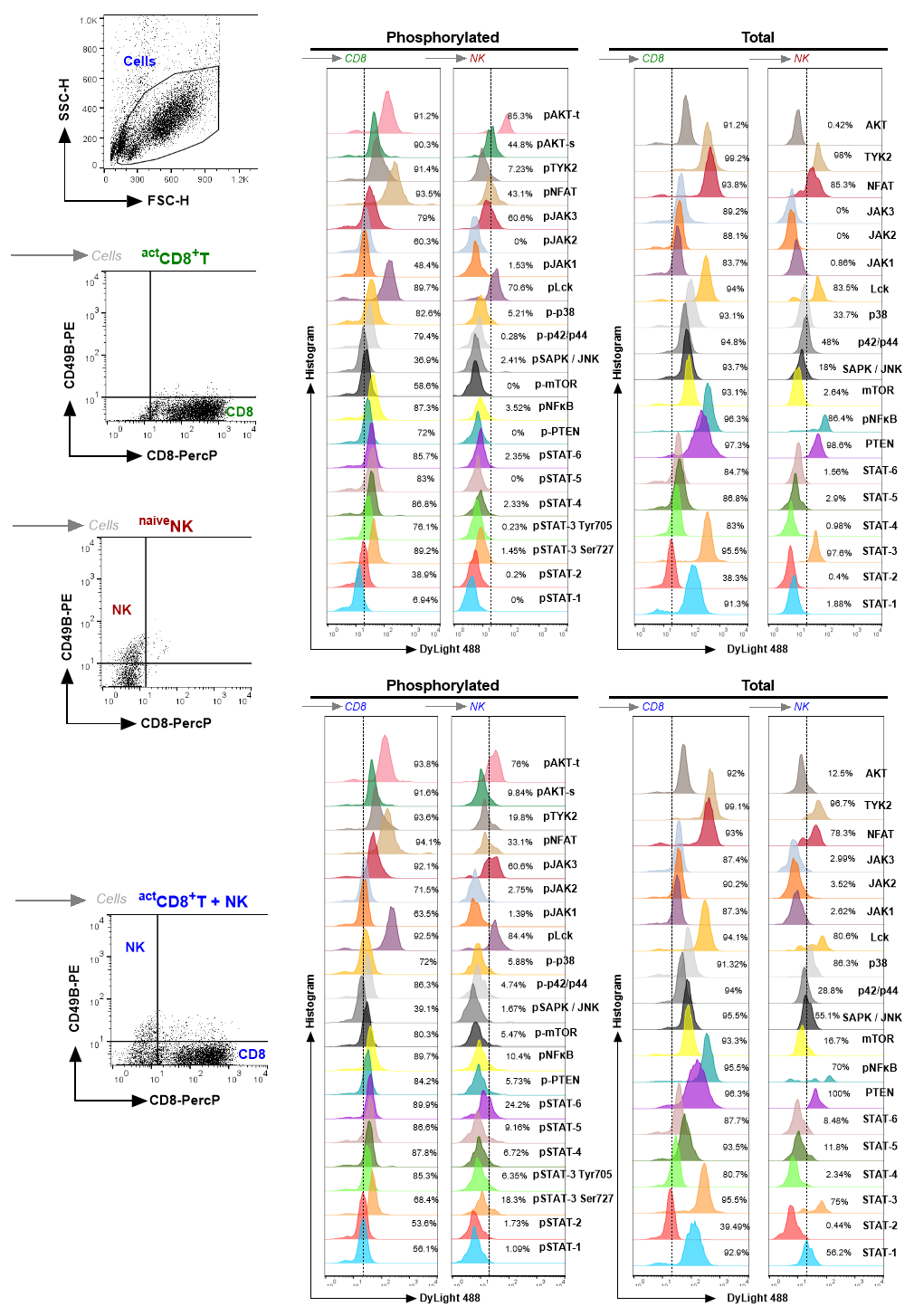


**SUPPLEMENTARY FIGURE S6.** Flow cytometry analysis strategy for phospho‑ and total‑protein profiling in CD8⁺T and NK cells. Cells were first gated by FSC/SSC and selected into CD8⁺ and NK subsets.


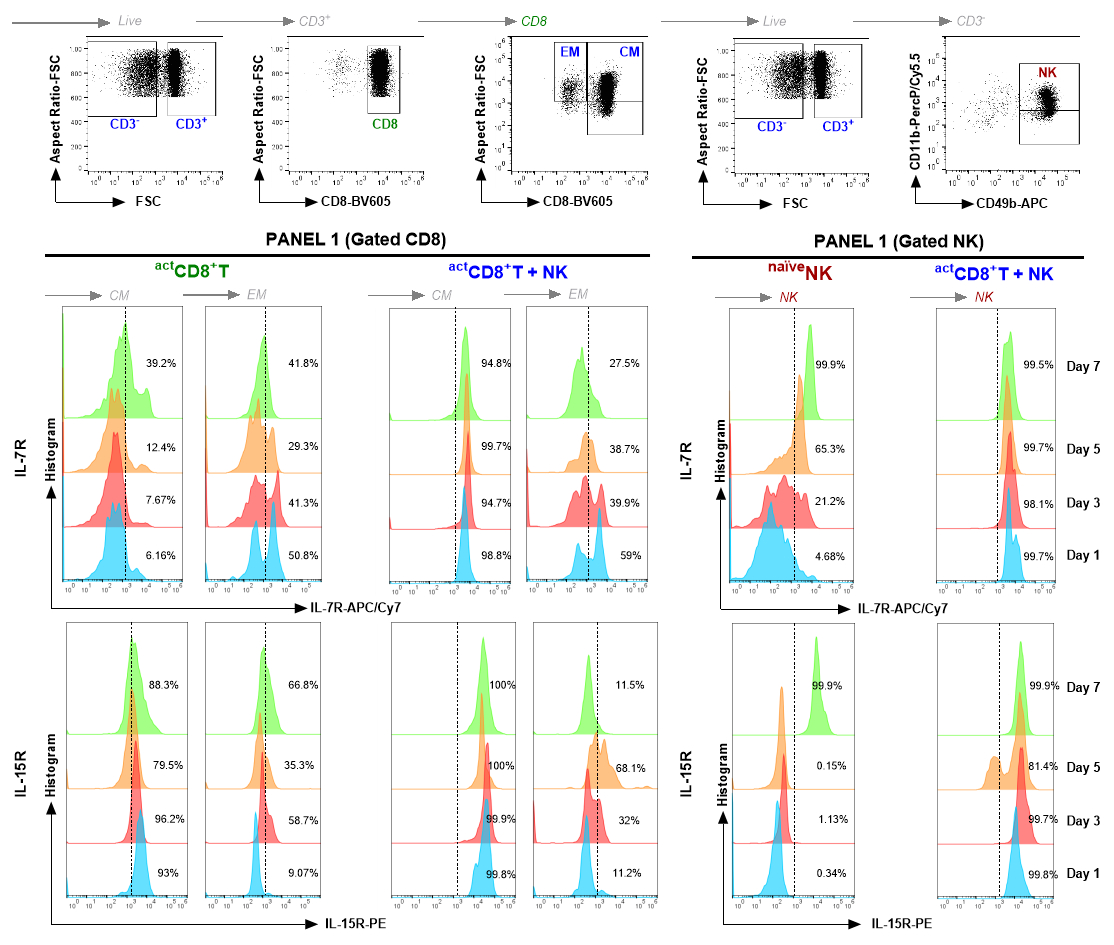


**SUPPLEMENTARY FIGURE S7.** FSC/SSC selected live lymphocytes, then gated into CD3⁺ versus CD3⁻ populations. CD3⁺CD8⁺ T cells were further subdivided into central memory (CM; CD62L⁺CD44⁺) and effector memory (EM; CD62L⁻CD44⁺) subsets, while NK cells were identified as live CD3⁻CD11b⁺CD49b⁺ subsets. Histograms show IL‑7R (upper panels) and IL‑15R (lower panels) expression on CM and EM CD8⁺ T cells, dual CD8⁺NK cells, and naïve NK cells at days 1, 3, 5, and 7. Vertical dotted lines mark positivity thresholds, and percentages denote the frequency of receptor‑positive cells. Data are representative of three independent experiments.


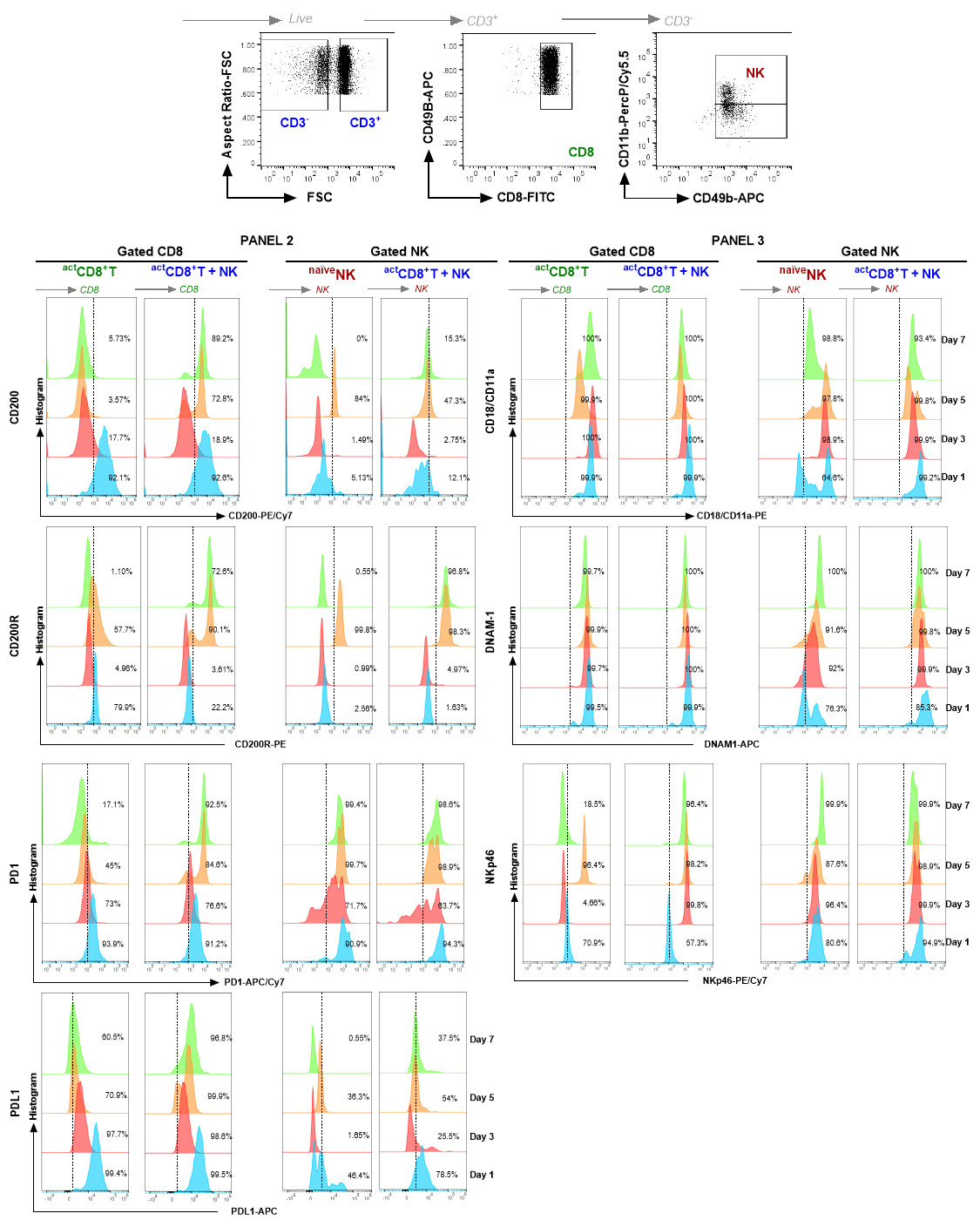


**SUPPLEMENTARY FIGURE S8.** Live lymphocytes were selected by FSC versus aspect‐ratio SSC, then gated on T cells (CD3⁺CD8⁺) and NK cells (CD3⁻CD11b⁺CD49b⁺). For Panel 2, surface expression of CD200, CD200R, PD‑1, and PD‑L1 was assessed on activated CD8⁺T cells cultured alone (T) or with NK cells (T+NK) and on naïve NK cells cultured alone or with CD8⁺T cells, at days 1, 3, 5 and 7. The same four populations and time points were analyzed for adhesion and co‑activation markers CD18/CD11a, DNAM‑1, and NKp46 in Panel 3. Data are representative of three independent experiments.


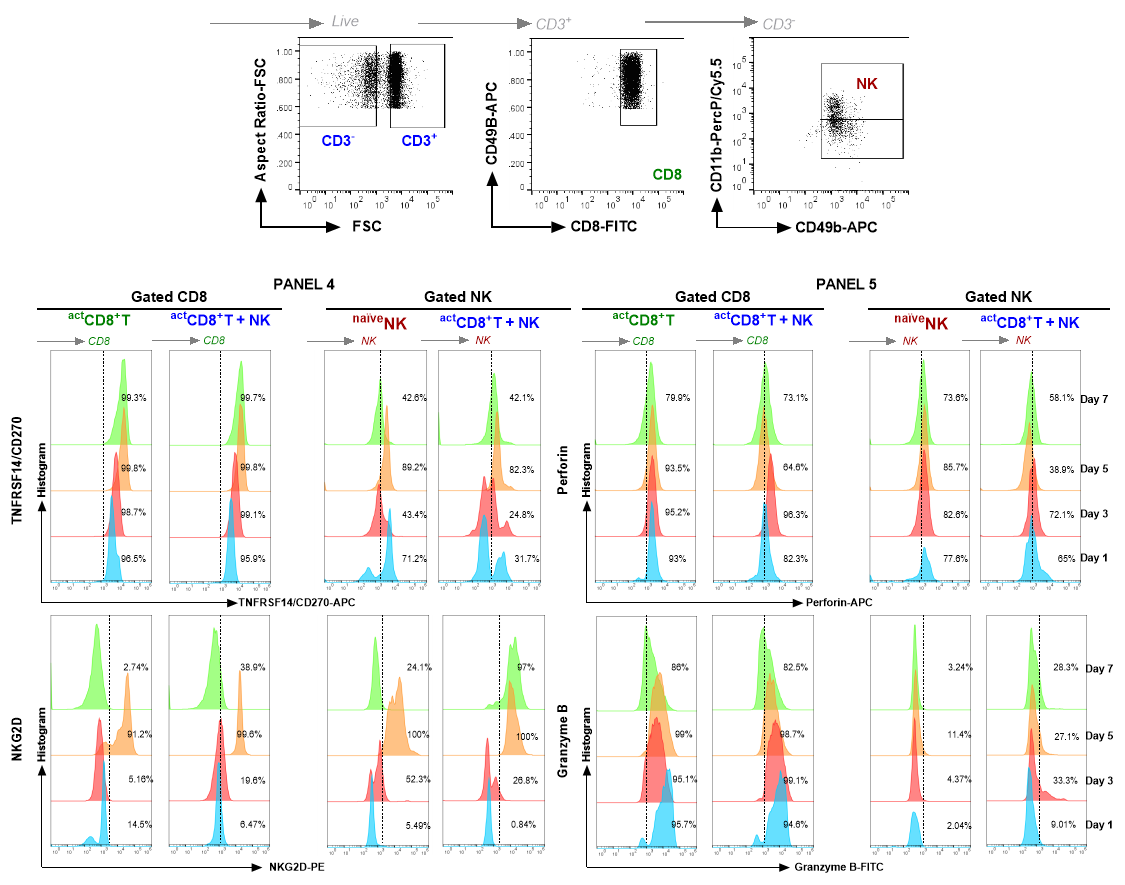


**SUPPLEMENTARY FIGURE S9.** Live lymphocytes were selected by FSC versus aspect‐ratio SSC, then gated on T cells (CD3⁺CD8⁺) and NK cells (CD3⁻CD11b⁺CD49b⁺). For Panel 4, surface expression of TNFRSF14/CD270 and NKG2D was assessed on activated CD8⁺T cells cultured alone (T) or with NK cells (T+NK) and on naïve NK cells cultured alone or with CD8⁺T cells at days 1, 3, 5 and 7. The same four populations and time points were analyzed for cytotoxicity molecules perforin and granzyme B Panel 5. Data are representative of three independent experiments.


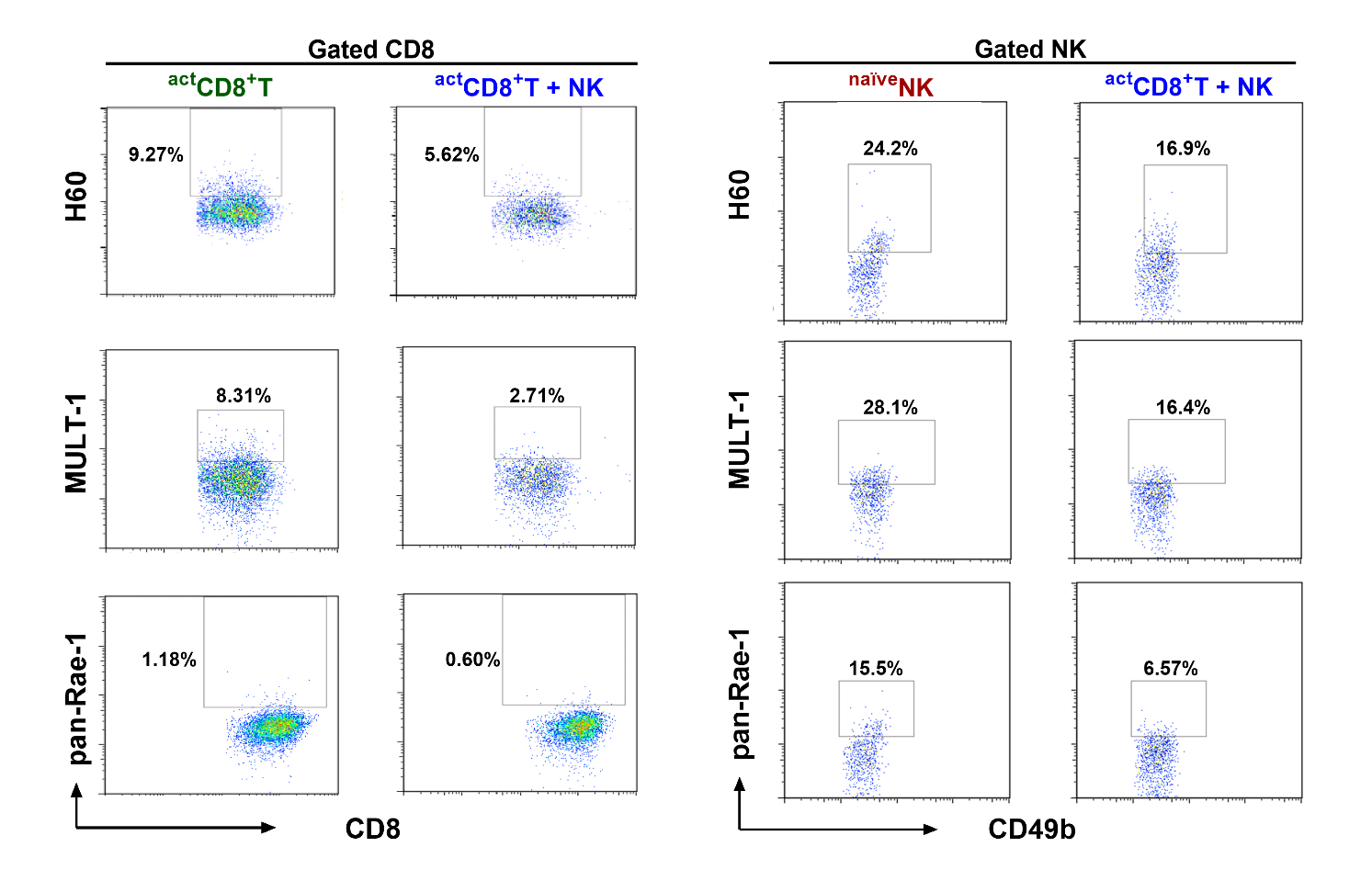


**SUPPLEMENTARY FIGURE S10.** Expression of NKG2D ligands, MULT-1, H60, and pan Rae-1 molecules on ^act^CD8^+^T cells and ^naïve^NK in Monoculture or at 36 h of Co-culture. ^act^CD8^+^T cells (0.5 x 10^6^) were co-cultured with ^naïve^CD49b^+^ (DX5^+^) NK cells (0.5 x 10^6^) on fibronectin (10 μg/mL) pre-coated plates.
