# Supplementary material for: CD8+ T–NK cell crosstalk establishes preemptive immunosurveillance to eliminate antigen–escape tumors
Source: Front Immunol. 2025 Sep 22;16:1593913. doi: 10.3389/fimmu.2025.1593913 (PMC12497863; doi:10.3389/fimmu.2025.1593913)
Supplement: Supplementary file 2 [file DataSheet2.docx]

Supplementary Material

# SUPPLEMENTARY VIDEOS

## Supplementary video legends

**SUPPLEMENTARY VIDEO S1.** Representative time-lapse confocal microscopy of co-cultured NK cells and CD8⁺T lymphocytes, illustrating dynamic nanotube formation and pseudopodial projections and invaginations that mediate continuous, bidirectional exchange of cytoplasmic molecules and membrane fragments between the two cell types. Please refer to *Video S1.*

**SUPPLEMENTARY VIDEO S2.** Representative time‑lapse confocal microscopy of CD8⁺T cell–NK cell crosstalk illustrating membrane exchange. CD8⁺T cells (red membrane label) and NK cells (purple membrane label) were co‑cultured and imaged to capture the transfer of membrane fragments (magenta) at the immune synapse. Please refer to *Video S2.*
