# Supplementary material for: CD8+ T–NK cell crosstalk establishes preemptive immunosurveillance to eliminate antigen–escape tumors
Source: Front Immunol. 2025 Sep 22;16:1593913. doi: 10.3389/fimmu.2025.1593913 (PMC12497863; doi:10.3389/fimmu.2025.1593913)
Supplement: Supplementary file 3 [file DataSheet3.docx]

Supplementary Material

# SUPPLEMENTARY TABLES

| SUPPLEMENTARY TABLE S1. *In silico* analysis for Ligand : Receptor pairs associated with CD8^+^T–NK crosstalk | | | | | | | | | |
| --- | --- | --- | --- | --- | --- | --- | --- | --- | --- |
| Ligand | | Receptor | | Transcripts Threshold | | ≥2 TPM | | ≥10 TPM | |
| ^act^CD8^+^T  Schmeidel (TPM) | **^naive^NK Schmeidel (TPM)** | **^act^CD8^+^T**  **Schmeidel (TPM)** | **^naive^NK Schmeidel (TPM)** | **≥2 TPM** | **≥10 TPM** | **Ligand** | **Receptor** | **Ligand** | **Receptor** |
| <1 | >2 | 2 | >2 | **YES** |  | WNT1 | FZD3/LRP5 |  |  |
|  |  | >5 | >3 | **YES** |  | WNT1 | FZD6/LRP5 |  |  |
| 3 |  | 2 | >2 | **YES** |  | WNT10A | FZD3/LRP5 |  |  |
|  |  |  | >2 | **YES** |  | WNT10A | FZD4/LRP5 |  |  |
|  |  | >5 | >3 | **YES** |  | WNT10A | FZD6/LRP5 |  |  |
| >3 | >12 | 2 | >2 | **YES** |  | WNT10B | FZD3/LRP5 |  |  |
|  |  |  | >2 | **YES** |  | WNT10B | FZD4/LRP5 |  |  |
|  |  | >5 | >3 | **YES** |  | WNT10B | FZD6/LRP5 |  |  |
|  |  | 30 | 200 | **YES** | **YES** | NAMPT | ITGA5/ITGB1 | NAMPT | ITGA5/ITGB1 |
| >40 | >40 | 10 |  | **YES** | **YES** | VEGFB | FLT1 | VEGFB | FLT1 |
|  | 40 | 3 | <2 | **YES** |  | TNFSF12 | TNFRSF12A |  |  |
| >4 | >15 | 70 | 5 | **YES** | **YES** | TNFSF10 | TNFRSF10A | TNFSF10 | TNFRSF10A |
| 100 | 5 | 20 | 60 | **YES** | **YES** | TNF | TNFRSF1A | TNF | TNFRSF1A |
|  |  | 300 | 150 | **YES** | **YES** | TNF | TNFRSF1B | TNF | TNFRSF1B |
| 8 | 75 | 6 | 9 | **YES** |  | TIGIT | PVR |  |  |
|  |  | 30 | 200 | **YES** |  | THBS1 | ITGA3/ITGB1 |  |  |
|  |  | >12 | >12 | **YES** |  | THBS1 | ITGAV/ITGB3 |  |  |
|  |  | 7 |  | **YES** |  | THBS1 | SDC4 |  |  |
|  |  | 150 | >200 | **YES** |  | THBS1 | CD47 |  |  |
|  |  | 30 | 200 | **YES** |  | THBS3 | ITGA3/ITGB1 |  |  |
|  |  | >12 | >12 | **YES** |  | THBS3 | ITGAV/ITGB3 |  |  |
|  |  | 7 |  | **YES** |  | THBS3 | SDC4 |  |  |
|  |  | 150 | >200 | **YES** |  | THBS3 | CD47 |  |  |
|  |  | >700 | 200 | **YES** | **YES** | TGFB1 | TGFbR1/TGFbR2 | TGFB1 | TGFbR1/TGFbR2 |
|  |  | >20 | 5 | **YES** | **YES** | TGFB1 | ACVR1B/TGFbR2 | TGFB1 | ACVR1B/TGFbR2 |
|  |  | >700 | 200 | **YES** | **YES** | TGFB3 | TGFBR1/TGFBR2 | TGFB3 | TGFBR1/TGFBR2 |
|  |  | >20 | 5 | **YES** | **YES** | TGFB3 | ACVR1B/TGFBR2 | TGFB3 | ACVR1B/TGFBR2 |
|  |  | >8 | >8 | **YES** |  | SEMA7A | PLXNC1 |  |  |
| 200 | 100 |  | 30 | **YES** | **YES** | SEMA4D | CD72 | SEMA4D | CD72 |
| 100 | >500 | 3 | 1 | **YES** |  | SELPLG | SELP |  |  |
|  |  | 1700 | 400 | **YES** | **YES** | SELPLG | SELL | SELPLG | SELL |
| 7 | 5 | 2 | 2 | **YES** |  | INSL3 | RXFP4 |  |  |
| 8 | 15 | 8 | 15 | **YES** |  | PECAM1 | PECAM1 |  |  |
| 8 |  |  | >40 | **YES** |  | PDGFA | PDGFRB |  |  |
| 140 | 5 | 15 |  | **YES** |  | CD274 | PDCD1 |  |  |
|  | 300 | 2 | 100 | **YES** |  | GZMA | F2R |  |  |
| 2 |  | 20 | 8 | **YES** |  | TNFSF4 | TNFRSF4 |  |  |
|  |  | 80 | >5 | **YES** |  | OSM | OSMR/IL6ST |  |  |
| 6 | 12 | 18 | 80 | **YES** | **YES** | DLL1 | NOTCH1 | DLL1 | NOTCH1 |
|  |  | 70 | 50 | **YES** | **YES** | DLL1 | NOTCH2 | DLL1 | NOTCH2 |
| 6 | 9 | 300 | 250 | **YES** |  | NECTIN1 | CD96 |  |  |
|  |  | 5 |  | **YES** |  | NECTIN1 | NECTIN3 |  |  |
| 5 |  | 6 | 9 | **YES** |  | NECTIN3 | NECTIN1 |  |  |
|  |  | 5 | 60 | **YES** |  | NECTIN3 | TIGIT |  |  |
|  | 80 | 4 | 6 | **YES** |  | NCAM1 | FGFR1 |  |  |
| 7 | 2 | 7 | 2 | **YES** |  | MPZL1 | MPZL1 |  |  |
|  | >4 | 7 |  | **YES** |  | MDK | SDC4 |  |  |
|  |  | 30 | 200 | **YES** |  | MDK | ITGA4/ITGB1 |  |  |
|  |  | 30 | 60 | **YES** |  | MDK | ITGA6/ITGB1 |  |  |
|  |  | 1400 | 350 | **YES** |  | MDK | NCL |  |  |
| 300 | 5 | 40 | 90 | **YES** | **YES** | TNFSF14 | TNFRSF14 | TNFSF14 | TNFRSF14 |
|  |  | 30 | 200 | **YES** |  | LAMA2 | ITGA1/ITGB1 |  |  |
|  |  | >2 | 1 | **YES** |  | LAMA2 | ITGA3/ITGB1 |  |  |
|  |  | 30 | 60 | **YES** |  | LAMA2 | ITGA6/ITGB1 |  |  |
|  |  | 900 | 70 | **YES** |  | LAMA2 | CD44 |  |  |
|  |  | 2 | 2 | **YES** |  | LAMA2 | SV2A |  |  |
|  |  | 8 | 15 | **YES** |  | LAMA2 | DAG1 |  |  |
|  |  | 10 | 900 | **YES** | **YES** | F11R | ITGAM/ITGB2 | F11R | ITGAM/ITGB2 |
|  |  | 10 | 25 | **YES** | **YES** | F11R | F11R /F11R | F11R | F11R /F11R |
|  |  | 24 | 2 | **YES** | **YES** | F11R | JAM3/JAM3 | F11R | JAM3/JAM3 |
|  |  | 10 | 900 | **YES** | **YES** | JAM3 | ITGAM/ITGB2 | JAM3 | ITGAM/ITGB2 |
|  |  | 10 | 25 | **YES** | **YES** | JAM3 | F11R /F11R | JAM3 | F11R /F11R |
|  |  | 24 | 2 | **YES** |  | JAM3 | JAM3 |  |  |
| 10 | 900 | 20 | 90 | **YES** | **YES** | ITGAL/ITGB2 | CD226 | ITGAL/ITGB2 | CD226 |
|  |  | 40 | 20 | **YES** | **YES** | ITGAL/ITGB2 | ICAM1 | ITGAL/ITGB2 | ICAM1 |
|  |  | 35 | 40 | **YES** | **YES** | ITGAL/ITGB2 | ICAM2 | ITGAL/ITGB2 | ICAM2 |
|  |  | 200 | 1800 | **YES** | **YES** | IL15 | IL15RA/IL2RB | IL15 | IL15RA/IL2RB |
|  |  | 90 |  | **YES** | **YES** | IFNG | IFNGR1/IFNGR2 | IFNG | IFNGR1/IFNGR2 |
|  |  | 10 | 900 | **YES** | **YES** | ICAM1 | ITGAX/ITGB2 | ICAM1 | ITGAX/ITGB2 |
|  |  | 10 | 1200 | **YES** | **YES** | ICAM1 | ITGAL/ITGB2 | ICAM1 | ITGAL/ITGB2 |
|  |  |  | 500 | **YES** | **YES** | ICAM1 | ITGAM/ITGB2 | ICAM1 | ITGAM/ITGB2 |
|  |  | 20 | 210 | **YES** | **YES** | ICAM1 | SPN | ICAM1 | SPN |
|  |  | 10 | 900 | **YES** | **YES** | ICAM2 | ITGAX/ITGB2 | ICAM2 | ITGAX/ITGB2 |
|  |  |  | 500 | **YES** | **YES** | ICAM2 | ITGAM/ITGB2 | ICAM2 | ITGAM/ITGB2 |
|  |  | 5 | 12 | **YES** |  | GDF11 | TGFBR1/ACVR2A |  |  |
|  |  | 5 | 2 | **YES** |  | GDF11 | TGFBR1/ACVR2B |  |  |
|  |  | 5 | 2 | **YES** |  | GDF11 | ACVR1B/ACVR2B |  |  |
| 2 | 25 | 1000 | 1300 | **YES** | **YES** | LGALS9 | PTPRC | LGALS9 | PTPRC |
|  |  | 5 | 90 | **YES** |  | LGALS9 | HAVCR2 |  |  |
|  |  | 900 | 70 | **YES** | **YES** | LGALS9 | CD44 | LGALS9 | CD44 |
| 4 |  | 4 | 5.5 | **YES** |  | FGF9 | FGFR1 |  |  |
| 5 | 40 | 45 | 20 | **YES** | **YES** | FASLG | FAS | FASLG | FAS |
| 4 | 2 | 1.5 | 20 | **YES** |  | EFNA1 | EPHA4 |  |  |
|  |  | 2 |  | **YES** |  | EFNA1 | EPHA1 |  |  |
| 100 | 300 | 2 | 3 | **YES** |  | CD99 | PILRA |  |  |
| 300 | 200 | 6 | 9 | **YES** |  | CD96 | NECTIN1 |  |  |
| 350 | 60 | 13 |  | **YES** | **YES** | CD6 | ALCAM | CD6 | ALCAM |
| 400 | 220 |  | 450 | **YES** | **YES** | CD48 | CD244 | CD48 | CD244 |
|  |  | 30 | 200 | **YES** | **YES** | CD40LG | ITGA5/ITGB1 | CD40LG | ITGA5/ITGB1 |
|  |  | 10 | 900 | **YES** | **YES** | CD40LG | ITGAM/ITGB2 | CD40LG | ITGAM/ITGB2 |
| 3 | 5 | 2 |  | **YES** |  | ENTPD1 | ADORA2A |  |  |
|  |  | 8 | 2 | **YES** |  | ENTPD1 | ADORA2B |  |  |
| 20 | 90 | 6 | 9 | **YES** |  | CD226 | PVR |  |  |
| 6 | 120 |  | 2 | **YES** |  | CCL3 | CCR1 |  |  |
|  |  |  | 3 | **YES** |  | CCL3 | CCR5 |  |  |
| 70 | 2800 |  | 2 | **YES** |  | CCL5 | CCR1 |  |  |
|  |  | 7 |  | **YES** |  | CCL5 | CCR4 |  |  |
|  |  |  | 3 | **YES** |  | CCL5 | CCR5 |  |  |
| 120 |  | 40 | 90 | **YES** | **YES** | BTLA | TNFRSF14 | BTLA | TNFRSF14 |
|  |  | 5 | 12 | **YES** |  | BMP8B | ACVR1/ACVR2A |  |  |
|  |  | 5 | 2 | **YES** |  | BMP8B | ACVR1/ACVR2B |  |  |
|  |  | 6 | 17 | **YES** |  | BMP8B | ACVR1/BMPR2 |  |  |
| 150 | 125 | 2 | 250 | **YES** |  | BAG6 | NCR3 |  |  |
| 43 |  | 100 | 150 | **YES** | **YES** | APP | CD74 | APP | CD74 |
| 12 |  | 350 | 60 | **YES** | **YES** | ALCAM | CD6 | ALCAM | CD6 |
|  |  |  |  | **105** | **40** |  |  |  |  |

Supplementary Table S1: *In silico* database analysis shows 105 L : R pairs expressed in ^act^CD8^+^ and NK cells associated with T–NK crosstalk (*red highlighted*) or particularly relevant to immune functions (*blue highlighted*). The numbers at the bottom of the table indicate the total counts of interactions in each category.

| SUPPLEMENTARY TABLE S2. Survival analysis of mice bearing P511 or P1.204 tumors and administered with D_–7_ or D_+1_ adoptive CD8⁺T cell transfers | | | | | | | | |
| --- | --- | --- | --- | --- | --- | --- | --- | --- |
| Days post tumor | D_-7_ T - P511 | | D_+1_ T - P511 | | D_-7_ T - P1.204 | | D_+1_ T - P1.204 | |
| **0** | 100 |  | 100 |  | 100 |  | 100 |  |
| **26** |  |  |  |  |  |  | 89.474 | 7.041 |
| **27** |  |  |  |  |  |  | 84.211 | 8.365 |
| **29** | 100 |  | 100 |  | 83.333 | 8.794 | 63.158 | 11.06 |
| **30** |  |  |  |  | 72.222 | 10.557 | 57.895 | 11.327 |
| **33** |  |  |  |  | 50 | 11.785 | 52.632 | 11.455 |
| **34** |  |  |  |  | 11.111 | 7.407 | 31.579 | 10.664 |
| **35** |  |  |  |  | 0 | 0 | 26.316 | 10.102 |
| **36** |  |  |  |  |  |  |  |  |
| **41** |  |  | 90.476 | 6.406 |  |  |  |  |
| **46** |  |  | 803952 | 8.569 |  |  | 0 | 0 |
| **54** |  |  | 61.905 | 10.597 |  |  |  |  |
| **55** |  |  | 52.381 | 10.899 |  |  |  |  |
| **57** |  |  | 33.333 | 10.287 |  |  |  |  |
| **59** |  |  | 23.810 | 9.294 |  |  |  |  |
| **62** | 100 |  | 19.048 | 8.569 |  |  |  |  |
| **68** |  |  | 4.762 | 4.647 |  |  |  |  |
| **101** | 100 |  |  |  |  |  |  |  |
| Median survival | Undefined | | 57 | | 34.5 | | 34 | |

| *Log-rank (Mantel-Cox) test* | |
| --- | --- |
| Chi-square | 101.4 |
| df | 3 |
| P value | <0.0001 |
| P value summary | **** |
| Are the survival curves significantly different? | Yes |

Supplementary Table S2: Survival percentages and statistical analysis of mice bearing P511 or P1.204 tumors administered with adoptive CD8⁺T cells at different time points. Percentage of surviving mice over time following adoptive transfer of CD8⁺T cells at day –7 (D_–7_) or day +1 (D_+1_) relative to tumor injection. Median survival is indicated for each group. Survival curves were analyzed using the Log-rank (Mantel-Cox) test, which showed a significant difference among groups (χ² = 101.4, df = 3, p < 0.0001) study period.

| SUPPLEMENTARY TABLE S3. Average T cell counts per CFSE divisions | | | | | | | |
| --- | --- | --- | --- | --- | --- | --- | --- |
| Groups | D0 | D1 | D2 | D3 | D4 | D5 | D6 |
| **DC+Media** | 6111 | 264 | 45.5 | 20.7 | 21.3 | 18 | 20.2 |
| P511-10^2^ | 8746 | 319 | 70.3 | 189 | 284 | 209 | 83.1 |
| P511-10^3^ | 1506 | 192 | 485 | 785 | 1307 | 1169 | 285 |
| P511-10^4^ | 1632 | 213 | 940 | 1821 | 2934 | 2310 | 609 |
| P511-10^5^ | 136 | 65.8 | 931 | 3677 | 7768 | 3782 | 0 |
| P511+DC-10^2^ | 3868 | 54.3 | 54 | 107 | 179 | 113 | 54 |
| P511+DC-10^3^ | 1853 | 260 | 707 | 1117 | 1522 | 1381 | 664 |
| P511+DC-10^4^ | 1279 | 196 | 649 | 1262 | 1884 | 1665 | 781 |
| P511+DC-10^5^ | 20.6 | 32.2 | 78 | 651 | 2128 | 2484 | 312 |
| **DC+P1Ap  (10^-7^M)** | 149 | 200 | 944 | 2970 | 4094 | 3759 | 1714 |

Supplementary Table S3: Average T cell counts per division derived from the proliferation model in FlowJo. The table presents the average number of cells observed in each generation (Division 0 to Division 6) over time for different experimental groups. CD8⁺T cells were stimulated with different concentrations of P511 tumor cells (10² to 10⁵), either alone or in combination with dendritic cells (DCs). Additional groups included DCs treated with media only (negative control) and loaded with P1Ap peptide (10⁻⁷ M) as a positive control. Cell division profiles were analyzed using FlowJo’s proliferation model, which calculated generation-specific cell numbers based on CFSE dye dilution.

| SUPPLEMENTARY TABLE S4. Average T cell counts per CFSE dilution generation | | | | | | | |
| --- | --- | --- | --- | --- | --- | --- | --- |
| Groups | D0 | D1 | D2 | D3 | D4 | D5 | D6 |
| **Rag1^-/-^ B10.D2** P511-CLN | 17219 | 1549 | 259 | 53.5 | 14.3 | 6.01 | 11.8 |
| **Rag1^-/-^ B10.D2** P511-TDLN | **3240** | **168** | **726** | **1992** | **3087** | **0** | **1356** |
| **Rag1^-/-^ B10.D2** P1.204-CLN | 12876 | 736 | 86.7 | 25.4 | 6.47 | 52.8 | 12.4 |
| **Rag1^-/-^ B10.D2** P1.204-TDLN | 2420 | 154 | 23 | 9.80 | 32.3 | 11.4 | 12.7 |
| **Rag1^-/-^ B6**  P511-CLN | 4927 | 53.7 | 40.9 | 13.6 | 0 | 0 | 7.81 |
| **Rag1^-/-^ B6**  P511-TDLN | **131** | **67.2** | **89.2** | **191** | **408** | **202** | **20.5** |
| **Rag1^-/-^ B6**  P1.204-CLN | 16151 | 142 | 21.4 | 8.49 | 8.10 | 9.71 | 15.1 |
| **Rag1^-/-^ B6**  P1.204-TDLN | 6958 | 176 | 43.5 | 9.61 | 0 | 0 | 85.6 |

Supplementary Table S4: Representative table showing average cell counts per generation from the FlowJo proliferation analysis. Data corresponds to *Rag1*⁻^/^⁻ B10.D2 and *Rag1*⁻^/^⁻ B6 hosts bearing either P511 or P1.204 tumors. T cell proliferation varied by tumor type, lymph node location, and host background, with enhanced division observed in the P511-TDLN group from Rag1⁻^/^⁻ B10.D2 mice. The generational proliferation was calculated using CFSE dye-based dilution tracking and analyzed via FlowJo’s proliferation model.

**SUPPLEMENTARY TABLE S5.** **Summary statistics of Speed–Distance Index (SDI) values for interacting cell types.**

| SDI summary statistics | | | | | | |
| --- | --- | --- | --- | --- | --- | --- |
| Interaction type | *Count* | *Mean* | *SD* | *50%* | *75%* | *Max* |
| **NK–NK** | 273 | 0.0360 | 0.0841 | 0 | 0.0246 | 0.7839 |
| **NK–T** | 1275 | 0.1017 | 0.1327 | 0.0632 | 0.1298 | 0.9499 |
| **T–T** | 3711 | 0.0790 | 0.1041 | 0.0462 | 0.1133 | 1.8678 |

| Interaction type | *p-value* |
| --- | --- |
| **NK–T vs NK–NK** | <0.001 |
| **NK–T vs T–T** | <0.001 |
| **Kruskall-Wallis** | <0.001 |

Supplementary Table S5: Summary statistics of Speed–Distance Index (SDI) values for different immune cell interaction types. The table summarizes SDI values calculated for NK–NK, NK–T, and T–T cell interactions based on tracked cell motility and proximity over time.

**SUPPLEMENTARY TABLE S6.** **Statistical analysis of adhesion, stimulatory and effector molecule expression on CD8⁺T cells and NK cells over time in coculture.**

| **Gated CD8 (^act^CD8T^+^ vs. ^act^CD8^+^T + NK)** | | | | |
| --- | --- | --- | --- | --- |
| **Protein** | **Day 1** | **Day 3** | **Day 5** | **Day 7** |
| **CD200** | 0.9999 | 0.9993 | **<0.0001** | **0.0025** |
| **CD200R** | **0.0071** | >0.9999 | 0.0959 | **<0.0001** |
| **PD1** | 0.9712 | 0.9538 | **<0.0001** | **<0.0001** |
| **PDL1** | >0.9999 | 0.9998 | **<0.0001** | **<0.0001** |
| **CD18/CD11a** | 0.9953 | 0.9997 | >0.9999 | 0.9997 |
| **DNAM-1** | **0.0050** | 0.2907 | 0.9939 | 0.9246 |
| **NKp46** | 0.9939 | 0.1814 | >0.9999 | 0.7625 |
| **TNFRSF14/CD270** | 0.8653 | 0.9700 | 0.9762 | 0.9102 |
| **NKG2D** | 0.9988 | 0.3920 | 0.9591 | 0.4180 |
| **Perforin** | **0.0041** | 0.9990 | **<0.0001** | 0.0877 |
| **Granzyme B** | 0.3692 | **0.0135** | 0.9789 | 0.0793 |

| **Gated NK (^naive^NK vs. ^act^CD8^+^T + NK)** | | | | |
| --- | --- | --- | --- | --- |
| **Protein** | **Day 1** | **Day 3** | **Day 5** | **Day 7** |
| **CD200** | 0.9055 | >0.9999 | 0.4477 | *0.0545* |
| **CD200R** | >0.9999 | 0.9961 | >0.9999 | **<0.0001** |
| **PD1** | 0.8700 | 0.3277 | >0.9999 | 0.9825 |
| **PDL1** | **0.0245** | 0.0880 | 0.9290 | **0.0432** |
| **CD18/CD11a** | *0.0578* | 0.9914 | 0.9901 | 0.9960 |
| **DNAM-1** | **0.0005** | **0.0007** | **<0.0001** | 0.9285 |
| **NKp46** | 0.9646 | 0.9984 | 0.5498 | 0.8465 |
| **TNFRSF14/CD270** | **0.0157** | 0.7098 | 0.9410 | 0.2055 |
| **NKG2D** | 0.9920 | 0.5227 | >0.9999 | 0.4553 |
| **Perforin** | **<0.0001** | **<0.0001** | **<0.0001** | **0.0017** |
| **Granzyme B** | **0.0003** | 0.8363 | **<0.0001** | **<0.0001** |

Supplementary Table S6: Statistical comparison of adhesion, stimulatory and effector molecule expression on CD8⁺T cells and NK cells over time in coculture. Table presents *p*-values from comparative analyses of protein expression across four time points (Days 1, 3, 5, and 7) during coculture.
*Top Panel*: Expression levels in activated CD8⁺T cells alone versus CD8⁺T cells cocultured with NK cells. Significant differences emerged for CD200, CD200R, PD-1, PD-L1, Perforin, and Granzyme B.
*Bottom Panel*: Comparison of naïve NK cells versus NK cells cocultured with activated CD8⁺T cells. Significant shifts were detected in PD-L1, DNAM-1, TNFRSF14/CD270, CD200R, Perforin, and Granzyme B.
